# Supplementary material for: What Drives Caterpillar Guilds on a Tree: Enemy Pressure, Leaf or Tree Growth, Genetic Traits, or Phylogenetic Neighbourhood?
Source: Insects. 2022 Apr 8;13(4):367. doi: 10.3390/insects13040367 (PMC9029432; doi:10.3390/insects13040367)
Supplement: Supplementary file 1 [file insects-13-00367-s001.zip › insects-1629574-supplementary.pdf]

Supplementary File S1

Molecular genetic analysis of oaks *Quercus petraea* and *Q. robur*

Introduction

In Central Europe, oaks are represented by two main species: pedunculate (*Quercus robur* L.) and sessile oak (*Q. petraea* (Matt.) Liebl.; [1,2]. Both species of oak may co-occur as a main component of temperate deciduous forests, despite their ecological differences, with tolerance to dry (*Q. petraea*) or wet (*Q. robur*) sites [3]. In addition to their undoubted cultural, industrial, and landscape significance, oaks support vast numbers of other species that depend on them for food [4,5].

Quercus petraea and *Q. robur* are highly outcrossing species with complex patterns of gene flow. Pollen and seed-mediated gene flow are bi-modal. Oaks seeds do not only spread locally [6,7], but also over distances of several kilometers or more, and pollen is dispersed widely by wind [8-12]. Intra- and interspecific gene flow varies depending on population density, variation among individuals in survival and reproduction, and geographic and environmental heterogeneity [13]. Adding to the complexity of the movement of genes is the fact that there is also human-assisted relocation of genotypes within the frame of the management and conservation of forests [14]. Nevertheless, low genetic differentiation between oak species and populations, high genetic diversity, and a low level of inbreeding detected so far support the concept of extensive gene flow and low genetic drift effects [15-21].

Frequent hybridization and backcrossing of hybrids with parental species (interspecific gene flow) can lead to genome size changes (including the gain or loss of DNA), shapes the community complex and structure [5,22], and blurred taxonomic boundaries of species. The taxonomic rank of oaks is sometimes questioned due to some morphological intermediacy, differences among trees in the ability to hybridize, a discrepancy between morphological and other characters, and low heritability values for secondary metabolites and leaf morphological traits [16,23-26]. Therefore, in the context of molecular data most reports of species-specific variants in oaks are based on allele frequency [18,19,27,28]. In closely related species *Q. petraea* and *Q. robur,* although two clades (“petraea and “robur) are obtained, it was not possible to assign all individuals to the morphological species to which they allegedly belonged, e.g., [29]. Recently, as Hipp et al. [30] showed, phylogenetic relationships between two species based on restriction-site associate sequencing are poorly supported. Such little inter-specific variation is explained by (1) micro-site selection which counteracts the homogenizing effect of interspecific gene flow and incomplete lineage sorting [21,31], (2) shared polymorphism [19], and/or (3) gene flow which counteracts genetic drift and the accumulation of species-specific alleles. Nevertheless, Muir and Schloetterer [19] proposed a set of microsatellite markers that statistically differentiated two species. This set was restricted to three 'species-discriminant' loci by Neophytou et al. [32]. These markers may

represent regions of the genome affected by a directional selection that maintains species identity [21]. However, this prediction needs to be confirmed with tests that include widely distributed populations.

Studies on DNA ploidy and karyotype have been undertaken to understand the structure of the oak genome. Oaks are diploid, 2n = 2x = 24 [33], but polyploids and trees with small supernumerary chromosomes have also been reported [24,34-40]. The nuclear DNA contents for *Q. petraea* and *Q. robur* have been assessed by Olszewska and Osiecka [41], Greilhuber [42], Favre and Brown [43], Ohri and Ahuja [44], and Dzialuk et al. [38], but values vary widely depending on the methodology used and the eco-geographical origin of trees [45]. Data to date do not indicate species-specific differences in DNA content and C-banding patterns [38,44]. Nevertheless, intrapopulation and interpopulation variation on a broad geographical scale has been found [38,45].

In two field groups of *Q. petraea* occurring at spatially distant stands we explore genetic variation and structure based on microsatellite markers which became the markers of choice due to their high polymorphism, informativeness, and co-dominance, e.g. [46,47]. We also performed a survey of genome size variation in *Q. petraea* to assess the intraspecific variation in DNA amounts and evaluate the frequency with which polyploid individuals occur. We employed propidium iodide flow cytometry to meet the objective, a fast and accurate method that detects minor differences in DNA content [43,45,48,49]. One analyzed field population of *Q. petraea* occurs at a site where there is also *Q. robur*. Because all downstream processing relies on how trees are initially assigned to putative species, we employed an agglomerative clustering method (neighbor-joining) based on microsatellite loci recognized as highly statistically differentiating both species [19,32]. Evaluation of oaks from a (cyto)genetic point of view was an essential part of evolutionary-ecological studies devoted to insect adaptations to selected trees as a function of isolation from neighboring trees.

Material and methods

Plant material

Assessment of genetic variation, based on SSR markers, was performed for 54 oak trees sampled in two locations in Puszcza Zielonka Landscape Park, to the northeast of the city of Poznań in Greater Poland Voivodeship, Poland. Details on the tree`s site are in Table S1.1. Initially, oak trees were assigned to species based on morphological traits. *Quercus petraea* has long petioles compared to the short petioles of *Q. robur*, and the former rarely have intercalary veins, which are common in *Q. robur* [8]. Individual variation in amplification efficiency of some loci resulted in a high number of missing values in the case of three trees that were excluded from the further analysis. Finally, a population genetic investigation was performed on 51 trees considering between 17 SSR and 12 SSR loci depending on the analysis. For

49 samples, cellular DNA content was obtained by flow cytometry to disclose patterns of genome size variations and ploidy of the measured cell populations (Table S1.1).

Table S1.1. List of accessions of *Q. petraea* and *Q. robur* used for genetic diversity analysis based on SSR markers and cellular DNA content measured by flow cytometry (FCM); NA – missing values.

No	locality	Individual field ID_	Morphological identification	latitude	longitude	SSRs	FCM
1	2	10	*Q. petraea*	52.52066	17.09237	✓	NA
2	2	12	*Q. petraea*	52.5247	17.09093	✓	✓
3	2	13	*Q. petraea*	52.51511	17.09431	✓	✓
4	2	14	*Q. petraea*	52.52465	17.09167	✓	✓
5	2	15	*Q. petraea*	52.51556	17.09366	✓	✓
6	2	17	*Q. petraea*	52.51969	17.06731	✓	✓
7	2	22	*Q. petraea*	52.51443	17.05664	✓	✓
8	2	23	*Q. petraea*	52.5144	17.0578	✓	✓
9	2	24	*Q. petraea*	52.52544	17.05574	NA	✓
10	2	25	*Q. petraea*	52.52506	17.05419	✓	✓
11	2	26	*Q. petraea*	52.52464	17.05693	✓	✓
12	1	31	*Q. petraea*	52.54475	17.12317	✓	✓
13	1	32	*Q. petraea*	52.54494	17.12405	✓	✓
14	1	33	*Q. petraea*	52.54444	17.12281	✓	✓
15	1	1	*Q. petraea*	52.54257	17.14511	✓	✓
16	1	30	*Q. petraea*	52.53933	17.12985	✓	✓
17	1	34	*Q. petraea*	52.54627	17.12312	✓	✓
18	1	35	*Q. petraea*	52.5461	17.12331	✓	✓
19	2	36	*Q. petraea*	52.3328	17.0847	✓	✓
20	2	36+	*Q. petraea*	52.3328	17.0847	✓	NA
21	2	37	*Q. petraea*	52.3087	17.03196	✓	✓
22	2	37g	*Q. petraea*	52.3087	17.03196	✓	✓
23	1	2	*Q. petraea*	52.54324	17.14517	✓	✓
24	2	38	*Q. petraea*	52.3087	17.0319	✓	✓
25	2	38f	*Q. petraea*	52.3087	17.0319	✓	✓
26	1	39	*Q. petraea*	52.3355	17.08098	✓	✓
27	1	40	*Q. petraea*	52.3352	17.08072	✓	✓
28	1	41	*Q. petraea*	52.3358	17.08172	✓	✓
29	1	42	*Q. petraea*	52.3356	17.08179	✓	✓
30	1	3	*Q. petraea*	52.54252	17.14413	✓	✓
31	1	45	*Q. petraea*	52.3244	17.08754	✓	✓
32	1	45+	*Q. petraea*	52.3244	17.08754	✓	✓
33	2	43	*Q. petraea*	52.3116	17.0556	✓	✓
34	1	44	*Q. petraea*	52.53938	17.14528	✓	✓
35	1	4	*Q. petraea*	52.53989	17.14317	✓	✓
36	1	5	*Q. petraea*	52.53968	17.14378	✓	✓
37	1	27	*Q. petraea*	52.54068	17.12975	✓	✓
38	1	28	*Q. petraea*	52.54005	17.12907	✓	✓
39	1	45B	*Q. petraea*	52.3244	17.08754	NA	✓
40	1	45C	*Q. petraea*	52.3244	17.08754	NA	✓

No	locality	Individual field ID_	Morphological identification	latitude	longitude	SSRs	FCM
41	2	16	*Q. robur*	52.51987	17.06762	✓	✓
42	2	19	*Q. robur*	52.52422	17.05025	✓	NA
43	1	6	*Q. petraea*	52.53857	17.14676	✓	✓
44	1	29	*Q. petraea*	52.53954	17.13018	✓	✓
45	1	42b	*Q. petraea*	52.3356	17.08179	✓	✓
46	1	42+	*Q. petraea*	52.3356	17.08179	✓	✓
47	1	42f	*Q. petraea*	52.3356	17.08179	✓	✓
48	1	45A	*Q. petraea*	52.3244	17,08754	NA	✓
49	2	11	*Q. petraea*	52.51906	17.09286	✓	✓
50	2	20	*Q. robur*	52.51596	17.06296	✓	NA
51	2	21	*Q. robur*	52.5164	17.06314	✓	NA
52	1	30b	*Q. petraea*	52.53933	17.12985	✓	✓
53	1	34F	*Q. petraea*	52.54627	17.12312	✓	✓
54	2	37a	*Q. petraea*	52.3087	17.03196	✓	✓

Microsatellite analysis

DNA extraction and PCR amplification

For each tree, DNA was extracted from a small part of a winter bud using the Syngen Plant DNA Mini & Maxi extraction kit and following the manufacturer's protocol. The DNA samples and tissues are currently preserved at the Department of Genetics at Adam Mickiewicz University in Poznań, Poland.

All trees were genotyped using a set of microsatellites published by Kampfer et al. [50]; Steinkellner et al. [51] with Muir and Schloetterer [19] primers modifications. Primers for PCR reactions were synthesized commercially, with the 5′ end of each set's forward primer labeled with one fluorescent dye. Either 6-FAM (blue), VIC (green), NED (yellow) or PET (red)]. 6-FAM were used for (36f, 102f, ssrQpZag1/5, ssrQpZAG7, ssrQrZag30, ssrQrZag96, ssrQrZag112), VIC (ssrQpZag15, ssrQpZag104, ssrQpZag108; ssrQrZag11, and ssrQrZag20, ssrQpZag46), NED (58f, ssrQpZag9, ssrQpZag 110, ssrQpZag 119, and ssQrZag 5), and PET (ssrQpZag58, ssrQpZag16); Applied Biosystems, Foster City, CA.

Multiplex PCR reactions were carried out in a 5 μl reaction volume containing 1x Type-It Microsatellite Kit (Qiagen), 0.2 μM forward primer, 0.2 μM fluorescent-labeled primer, and 1 μl (>5 to 20 ng) of DNA template, using a thermocycling profile of 1 cycle of 4 min at 94° C followed by 34 steps of 30 s at 94° C, 60 s at 45–57° C (depending on the primer set), and 60 s at 72° C, with a final step of 30 min at 72° C. DNA purity was verified by the absorbance ratio 260/280 nm, using for the subsequent analysis DNA samples with 1.8–2.0. The amplified alleles were separated on an ABI PRISM® 3130XL

(Applied Biosystems) with GeneScan® 600LIZ standard size. The amplicons were scored with Peak Scanner™ v.1.0 (Applied Biosystems).

Genetic relationship among collected samples

Provesti's genetic distances [52] between individuals based on 14 species-differentiated SSR loci [19,32] were employed for recognizing putative species (*Q. petraea* and *Q. robur*). The calculations were performed using the *poppr* R package [53] and plotted as a Neighbor-Joining (NJ) tree. The bootstrap method was used in the phylogenetic inference. The results of NJ clustering were compared to the morphological species identification. AMOVA, as implemented in GenAlEx v 6.3 [54], was also used to divide the total variance by morphologically divided species (*Q. petraea* and *Q. robur*) and spatial localities to rule out the possibility that detected variation reflects only a substructure within one species.

Assessing SSR data quality and power

The polymorphic information content (PIC) values were calculated for SSR markers using the formula of Liu, Que, and Pan [55]. Nuclear microsatellites are often characterized by a high frequency of null alleles that are not amplified in the PCR reaction and therefore not detected in heterozygotes [56,57]. The frequency of null alleles for each locus was estimated using *PopGenReport* [58] in R environment [59] according to Chakraborty et al. [60] and Brookfield [61] methods. To measure the probability of identity between genotyped individuals, a Multilocus Matches Analysis was performed as implemented in GenAlEx.

Genetic diversity statistics and deviations from Hardy-Weinberg equilibrium

Basic statistics for analyzed microsatellite data were evaluated in *poppr* (R), [53] and GenAlEx. Heterozygosity has been found to affect components of individual fitness, including survival, growth rate, or parasite resistance [62]; therefore, five individual heterozygosity estimates for analyzed oaks were calculated with R software using a *GENHET* function [63]. Hardy-Weinberg proportions were tested for each locus using exact tests suitable for a small sample size [64] in the R package *pegas* [65]. The threshold for statistical significance was set to *P*-value 0.05, with Bonferroni corrections for multiple comparisons.

Population structure and differentiation

Several approaches were undertaken to determine whether the analyzed group of *Q. petraea* have subpopulations and, if so, how many. Population genetic differentiation taking into account field localities was determined by estimating F_{ST} in R package *adegenet* [66]. This parameter was also calculated according to the ENA exclusion of null alleles method described by Chapuis and Estoup [57]

and implemented in the FreeNA program https://www1.montpellier.inra.fr/CBGP/software/FreeNA/ According to Waples and Gaggiotti [67], genetic differentiation is not negligible if F_{ST}≥0.05.

A Monte-Carlo assignment test was employed as implemented in R package *assignPOP* v.1.1.8 [68] to evaluate the assignment accuracy of individual trees to spatial locations and draw conclusions about the population structure. A Monte-Carlo (MC) cross-validation procedure [69,70] was used to split the data into training and test groups initially. In the MC, two levels of training individuals (0.5 and 0.7) from each spatial group and 0.10, 0.25, 0.5, and all loci were used as training data to perform the assignment test. The remainder of the individuals served as the test. A Support Vector Machine (model = "SVM) which has been shown to generate higher assignment accuracies than other models, was used to build predictive models [68]. Each combination of training data and the test dataset were iterated 30 times for a total of 240 assignment tests. This procedure allows evaluating variation in assignment accuracy and how different proportions of training individuals influenced the assignment results. The graphic was constructed in R, primarily using the package *ggplot2* [71].

The null hypothesis assuming a random distribution of genotypes in space was tested in a spatial autocorrelation analysis in GenAlEx, (no structure, autocorrelation coefficient, r=0). The program provides two nonparametric tests for statistical significance, such as bootstrap estimates (n = 999) of the 95% confidence interval (CI) around the value of r for each distance class size and random permutation estimates of the 95% CI around the null hypothesis of no positive genetic structure, r = 0. The significant spatial genetic structure is inferred if the calculated *r-value* falls outside the 95% confidence interval (r< or >0) [54].

The isolation-by-distance (IBD) hypothesis [72] is defined as a decrease in the genetic similarity among individuals/populations as the geographic distance between them increases. To test the hypothesis, the genetic relationship between pairwise oak tree individuals was estimated by Mantel tests as implemented in GenAlEx. The tests were performed on transformed and not transformed geographic distances [Log(1+GGD)] and means of genetic distance (*GD*) based on 12 loci. The significance level was computed through 999 permutations.

Recombination in the population of Q. petraea

A general idea of the importance of recombination in the analyzed spatially distant oak populations can be obtained by testing the null hypothesis of panmixia. Testing of this hypothesis is based on the assessment of linkage disequilibrium among loci (nonrandom association of alleles at different loci) [73]. If recombination dominates, LD can be ignored, whereas LD usually is observed when is population subdivision, loci are very closely linked, or when selection is strong [74,75]. The

association index among loci ($rbar_d$) was calculated in the R package *poppr* using the functions *pair.ia* and *resample.ia* [53].

Nuclear DNA content estimation by flow cytometry

We employed flow cytometry following the protocol of Galbraith et al. [76] and microsatellite profiles to determine DNA contents and disclose the DNA ploidy of the measured population. We based our DNA quantity assessment by flow cytometry on ten adult leaves taken on each tree representing *Q. petraea* and *Q. robur* (in total 49 trees x 10 biological replications), Table S1.1 In brief, to obtain nuclear suspensions, we first washed the fresh leaves five times in distilled water. Then we released nuclei from cells by chopping a fragment of a tree`s leaf (ca. a 1 cm^2) with leaf tissues of an internal standard – *Raphanus sativus* "Saxa (2C=1.11pg) in a Petri dish containing the ice-cold Galbraith's nuclear isolation buffer [0.1% Triton X-100, 1% (w/v) polyvinyl pyrrolidone 40000 and 5µl/ml 2-mercaptoethanol and 50 µl/ml RNAse (Sigma-Aldrich, USA)]. We used the standard for all measurements [77]. We filtered the resulting suspension through a 30µm nylon mesh (CellTrics. Sysmex Partec, Germany) and stained nuclei with 50 µl/ml propidium iodide (PI) (Sigma-Aldrich, USA) for 15 min. We kept the samples on ice and analyzed them immediately in a Cytomies FC500 (Beekman Coulter, USA) flow cytometer with a 488 nm laser and company software. At least 5,000 - 10,000 cells were measured per sample. We calculated the amount of DNA based on the 2C peak means of the sample and the internal standard according to the following formula:

$$Quercus\ species\ \text{ 2C nuclear DNA content (pg)} = \frac{Quercus\ sp.\ \text{ 2C peak mean}}{\text{Raphanus sativus "Saxa 2C peak mean}}\ \text{x}\ 1.11$$ (nuclear DNA content of the internal reference standard (pg)

To reject outliers in our measurements for each tree we performed two-sided Grubbs tests as implemented in the R package *outliers* v. 0.14 [78]. We analyzed DNA quantity data with nonparametric methods using Kruskal-Wallis one-way analysis of variance in TIBCO STATISTICA v. 13.3.0. We monitored the precision of the measurements by the Data coefficient of variation (CV%).

Results

Oak tree evaluation - tree identification based on species-differentiated SSR loci

In the selected set of SSR species-differentiated loci proposed by Muir and Schloetterer [19], genotyping was successful in 93% of samples (51 trees). These samples have 1.9% of missing values. The rest of the samples had high rates of missingness and were excluded from the subsequent analysis. The SSR data (14 loci) indicated a close relationship between *Q. petraea* and *Q. robur* individuals. Although trees morphologically classified as *Q. robur* form a single compact group (Figure S1.1), this group is nestled among *Q. petraea* trees. Moreover, it has very little bootstrap support suggesting that only a few

differences support that node. By reducing the number of species-specific diagnostic markers to, for example, 10, this coherent group of trees designated morphologically as *Q. robur* ceases to exist. Both species exhibited genetic variation. In the NJ tree, two *Q. robur* trees get bootstrap support above 65% (not shown), and two other *Q. petraea* trees had support above 75%. AMOVA revealed 22% of the genetic variation among individual trees, 72% within individuals, and 6% between species. Based on this set of markers, genetic differentiation between both species was low (F_{ST} = 0.058) but nevertheless highly significant (P< 0.001).

It was not possible to analyze our tree samples using only three markers as was deemed sufficient for the molecular identification of both species [32]. For two of the markers, 100% data were obtained. For the third marker, ssrQrZAG 30, no PCR product of the expected fragment length was received for 30% of the samples, possibly due to mutations of the primer binding site. This marker was therefore excluded from the subsequent analysis. The following population genetic analysis is limited to 47 individuals defined as *Q. petraea* and 12 SSR loci.

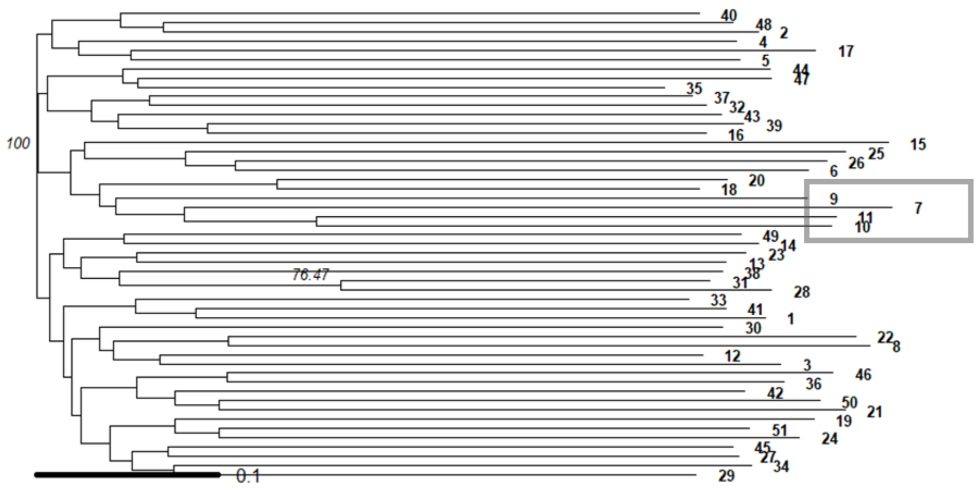

Figure S1.1. Provesti`s distance-based Neighbor-joining tree of *Quercus robur* and *Q. petraea* obtained from 14 species-differentiated SSR markers. Bootstrap values >70 are shown. The samples morphologically classified as *Q. robur* are in the grey frame; the remaining samples are identified based on morphology as *Q. petraea*.

Genetic data evaluation

From the 25 loci initially tested, we selected 17 loci for which we obtained the conclusive PCR product. Of these 17 loci, we excluded five loci from further analysis due to null alleles (frequency of null alleles ranged from 14% and 34%), see [79,80]. In the set of 12 loci analyzed, for nine loci, the frequency of null alleles did not exceed 3%, while for three loci, it ranged from 6% to 12% (loci 11, 20, and 108). Table S1.2 shows null allele frequency results calculated using Brookfield [61]. An average polymorphism information content value (PICavg.), calculated based on allele frequencies, ranged from

0.522 (locus 112) to 0.926 (locus 104) with an average equal 0.821 (Table S1.3). The high values indicate the potential utility of the SSR marker set to detect polymorphism among *Q. petraea* genotypes.

A total of 171 alleles and 85.4 effective alleles were detected. All of the SSR loci were highly polymorphic. Primer pairs for loci 102 and 104 detected the highest number of alleles (20). In contrast, the primer pair for locus 112 detected the fewest alleles (7). The allele frequency mean was 0.070, and frequencies varied from 0.011 to 0.667, meaning no fixed alleles (allele frequencies >90%) were found among the analyzed samples. Ninety-eight alleles (57.3%) presented rare allele frequencies (<5%).

Table S1.2. Null allele frequencies for 12 microsatellite loci in *Q. petraea* calculated using Brookfield (1996) method.

Locus	112	96	1_5	104	110	16	9	11	102	15	20	108
Observed frequency	0,023	-0,021	-0,017	0,030	0,003	0,040	0,036	0,066	0,072	0,040	0,124	0,090
Median frequency	0,017	-0,025	-0,024	0,025	-0,003	0,035	0,030	0,062	0,064	0,035	0,118	0,084
2.5th percentile	-0,050	-0,066	-0,067	-0,019	-0,054	-0,025	-0,027	-0,016	0,006	-0,033	0,038	0,017
97.5th percentile	0,099	0,024	0,030	0,082	0,060	0,107	0,105	0,160	0,134	0,118	0,210	0,168

Table S1.3. Descriptive statistics of the 12 SSR loci in *Quercus petraea* genotypes. Abbreviations: Na – Number of different alleles, Ne – Number of effective alleles 1/(Sum pi^2); AR – Allelic richness; H_E – Gene diversity; Ho – Observed heterozygosity; % difference between H_E and Ho; *P* value for deviations from the Hardy-Weinberg equilibrium calculated with the exact tests; Polymorphic Information Content (PIC).

Locus	Na	Ne	A_R	H_E	H_o	% difference	$P_{(HWE)}$ exact test	PIC value
112	7	2.094	6.616	0.522	0.489	6.427	0.377	0.522
96	13	6.011	12.539	0.748	0.681	8.931	0.109	0.834
1_5	11	6.133	9.895	0.882	0.674	23.580	0.691	0.837
104	20	13.521	19.248	0.896	0.739	17.489	0.019	0.926
110	15	5.375	13.936	0.834	0.872	-4.643	0.222	0.814
16	14	8.447	13.486	0.837	0.870	-3.896	0.318	0.882
9	11	6.745	10.581	0.926	0.870	6.098	0.430	0.852
11	14	3.952	12.576	0.814	0.809	0.667	0.000	0.747
102	20	11.137	18.436	0.882	0.809	8.293	0.000	0.910
15	13	3.962	11.533	0.852	0.787	7.574	0.491	0.748
20	16	8.464	14.742	0.747	0.638	14.545	0.000	0.882
108	17	9.596	15.438	0.910	0.783	14.019	0.011	0.896

Genetic diversity of Q. petraea *trees*

Based on the multilocus matches analysis, forty-seven unique genotypes of *Q. petraea* were found. All primers gave a probability of identical match by chance of 1.5E×10^{-17}. The 12 SSR markers exhibited a high level of diversity in *Q. petraea* samples, as shown by the values of the diversity statistics (Table S1.3). The mean expected heterozygosity (*He*) for the total sample was high *He* = 0.820 and ranged from 0.522 (locus112) to 0.926 (locus9). The mean observed heterozygosity (*Ho*) was lower than expected and equaled 0.752; varied from 0.489 to 0.872 for loci 112 and 110, respectively.

Because individual heterozygosity is associated with individual tree performance, five estimates of individual multi-locus heterozygosity (IH), considered representative of genome-wide inbreeding/outbreeding, are presented (Table S1.4). All multi-locus estimators are strongly correlated (Table S1.5), but some of our markers exhibited null alleles; therefore standardized heterozygosity based on the mean observed heterozygosity (Hs_obs) is the most reliable estimator of IH. The mean value of Hs_obs is equal to 0.999 ± SD 0.139.

Table S1.4. Individual heterozygosity estimates based on 12 SSR loci for 47 trees of *Q. petraea*: PHt - proportion of heterozygous loci (PHt) in an individual; Hs_exp - standardized heterozygosity based on the mean expected heterozygosity; Hs_obs - standardized heterozygosity based on the mean observed heterozygosity; IR - Internal relatedness; HL – homozygosity by locus; for a description of the parameters see [63].

NO	PHT	HS_OBS	HS_EXP	IR	HL
1	0.750	0.998	0.914	0.076	0.223
2	0.833	1.109	1.015	0.004	0.167
3	0.667	0.887	0.812	0.197	0.337
4	0.667	0.887	0.812	0.179	0.346
5	0.583	0.776	0.711	0.273	0.381
6	0.833	1.109	1.015	0.015	0.184
7	0.917	1.220	1.117	-0.053	0.083
8	0.833	1.109	1.015	-0.007	0.145
9	0.750	0.998	0.914	0.082	0.255
10	0.833	1.109	1.015	-0.030	0.144
11	0.833	1.109	1.015	0.073	0.143
12	0.750	0.998	0.914	0.102	0.273
13	0.667	0.887	0.812	0.240	0.306
14	0.750	0.998	0.914	0.044	0.212
15	0.583	0.776	0.711	0.305	0.388
16	0.583	0.776	0.711	0.259	0.398
17	0.750	0.998	0.914	0.090	0.218
18	0.636	0.845	0.782	0.234	0.379
19	0.917	1.220	1.117	-0.115	0.091
20	0.727	0.982	0.888	0.124	0.257
21	0.917	1.220	1.117	-0.053	0.090
22	0.833	1.109	1.015	0.002	0.170
23	0.750	0.998	0.914	0.075	0.234
24	0.750	0.998	0.914	0.107	0.252
25	0.917	1.220	1.117	-0.130	0.086
26	0.727	0.938	0.858	0.126	0.293
27	0.833	1.109	1.015	-0.026	0.165
28	0.667	0.887	0.812	0.168	0.341
29	0.500	0.665	0.609	0.373	0.471
30	0.750	0.998	0.914	0.059	0.218
31	0.833	1.109	1.015	-0.032	0.138
32	0.833	1.109	1.015	-0.042	0.129
33	0.917	1.220	1.117	-0.129	0.076

NO	PHT	HS_OBS	HS_EXP	IR	HL
34	0.833	1.109	1.015	-0.064	0.129
35	0.583	0.776	0.711	0.287	0.419
36	0.750	0.998	0.914	0.079	0.250
37	0.667	0.887	0.812	0.136	0.318
38	0.833	1.109	1.015	-0.037	0.172
39	0.833	1.109	1.015	0.018	0.181
40	0.583	0.776	0.711	0.294	0.425
41	0.800	1.058	0.993	-0.011	0.174
42	0.667	0.887	0.812	0.177	0.326
43	0.667	0.887	0.812	0.168	0.289
44	0.727	0.982	0.897	0.106	0.273
45	0.818	1.055	0.965	0.045	0.160
46	0.667	0.887	0.812	0.201	0.305
47	0.833	1.109	1.015	-0.036	0.129

Table S1.5. Pearson correlation between multi-locus estimators of individual heterozygosity based on 12 SSR loci for 47 trees of *Q. petraea*: PHt – the proportion of heterozygous loci (PHt) in an individual; Hs_exp - standardized heterozygosity based on the mean expected heterozygosity; Hs_obs - standardized heterozygosity based on the mean observed heterozygosity; IR - Internal relatedness; HL – homozygosity by locus. In bold values of the correlation coefficient significant at $P<0.05$.

	Mean	SD	PHt	Hs_obs	Hs_exp	IR	HL
PHt	0.752	0.105	1.000	**0.999**	**0.998**	**-0.974**	**-0.984**
Hs_obs	1.000	0.139	**0.999**	1.000	**0.999**	**-0.974**	**-0.983**
Hs_exp	0.916	0.128	**0.998**	**0.999**	1.000	**-0.975**	**-0.983**
IR	0.084	0.125	**-0.974**	**-0.974**	**-0.975**	1.000	**0.971**
HL	0.237	0.104	**-0.984**	**-0.983**	**-0.983**	**0.971**	1.000

Significant deviations from Hardy–Weinberg equilibrium were identified at two loci (loci 20, 102, alpha <0.0001) after Bonferroni corrections for multiple comparisons for the full data set. Based on the $rbar_d$ parameter, the null hypothesis of no linkage among markers cannot be rejected. This means also that the Wahlund effect due to unknown population stratification can be excluded (an important consequence of the Wahlund effect is linkage disequilibrium between different loci in our total sample). The observed $rbar_d$ equals 0.007 (P=0.293) and is located almost centrally of the re-sampled distribution (as expected from the unlinked loci, Figure S1.2).

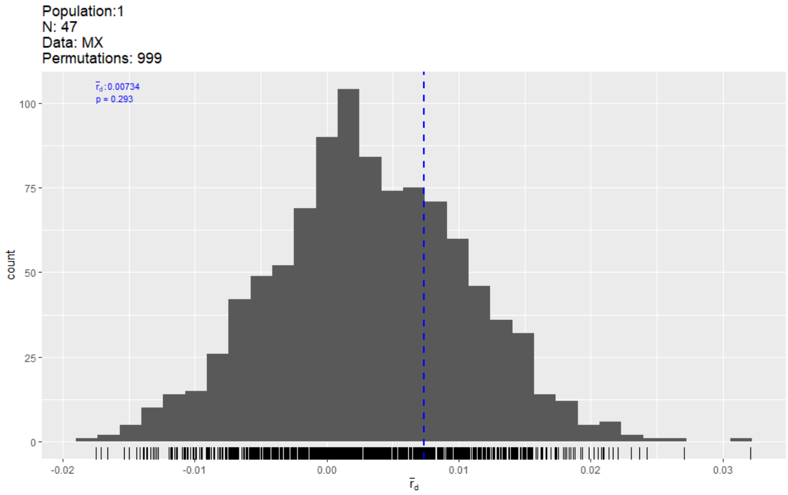

Figure S1.2. Observed index of association (rbar$_d$) on re-sample distribution (showing the variation of values observed at a given sample size) for 12 SSR loci analyzed in the total sample of individuals of *Q. petraea.*

Population structure

Wright`s F_{ST} was used to analyze the population differentiation for two sampling locations in the study. The genetic differentiation index (F_{ST}) is small F_{ST}= 0.002, but significant P=0.029, indicating high gene flow between two spatial populations. The ENA correction method for the positive bias induced by the presence of null alleles on the F_{ST} parameter means that F_{ST} did not differ substantially (F_{ST} not using ENA) = 0.00248; F_{ST} using ENA = 0.00323).

Assignment accuracies of Population1 are high, whereas for Population2 they are low. An individual tree's overall mean assignment accuracy for the field populations was relatively low (less than 65%) and slightly lower for 90% of training individuals than for 70%, regardless of the percentage of loci analyzed. (Figure S1.3).

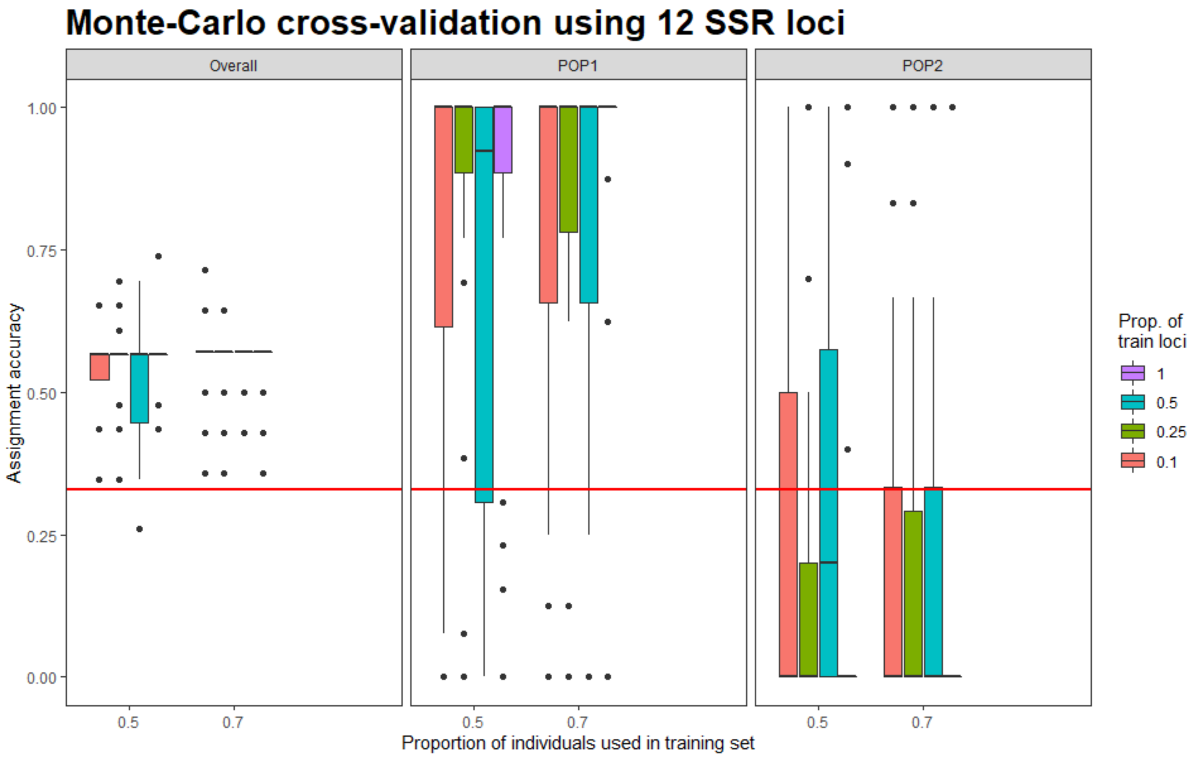

Figure S1.3. Assignment accuracies estimated via Monte–Carlo cross-validation and support vector machine methods, with a random sampling of two levels of training individuals (0.5, 0.7) crossed by four levels of high-F_{ST} training loci (0.1, 0.25. 0.5) and all loci for two spatial populations of *Q. petraea* based on 12 SSR loci. The left figure is the assignment on overall individuals. Top and bottom edges of the box are 25th and 75th percentiles; the ends of whiskers are the minimum and maximum of non-outliers; outliers are shown as black dots. Red horizontal lines indicate a null assignment rate (33%).

The spatial autocorrelation analysis does not show a continuous decrease in autocorrelation coefficient (*r*) with increasing geographic distances for the whole set of samples Figure S1.4, and Population1 (not shown). In Population2, however, the correlations drop with increasing distance and finally become negative, Figure S1.5. The positive genetic structure extends in the population to up to 1 km (P<0.05) and negative at 2.5 km (P=0.001).

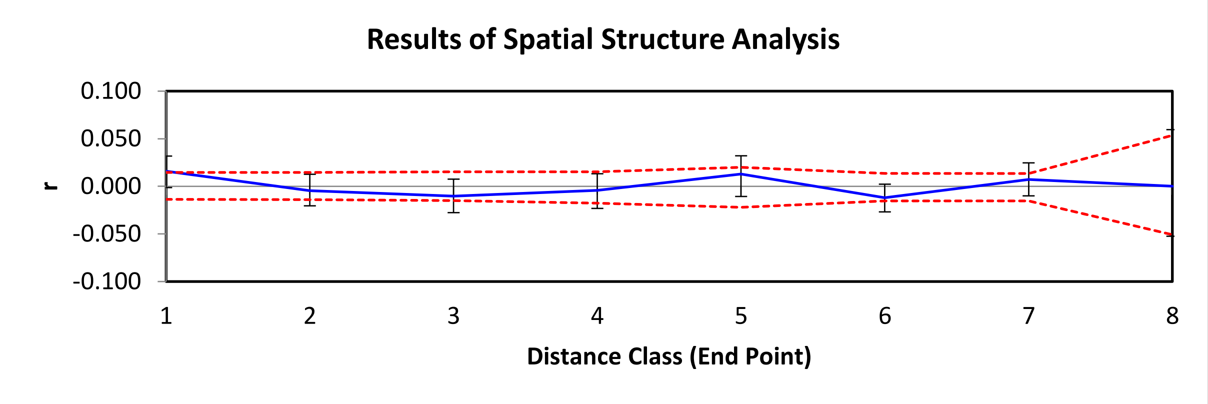

Figure S1.4. Global spatial autocorrelation for even distance classes (0-8 km) and their 95% confidence intervals (CI) for the total set of samples of *Q. petraea*. The 95% CI around the null hypothesis of no genetic structure is shown with dashed lines; error bars bound the 95% confidence interval about *r* as determined by 1000 bootstrap resampling.

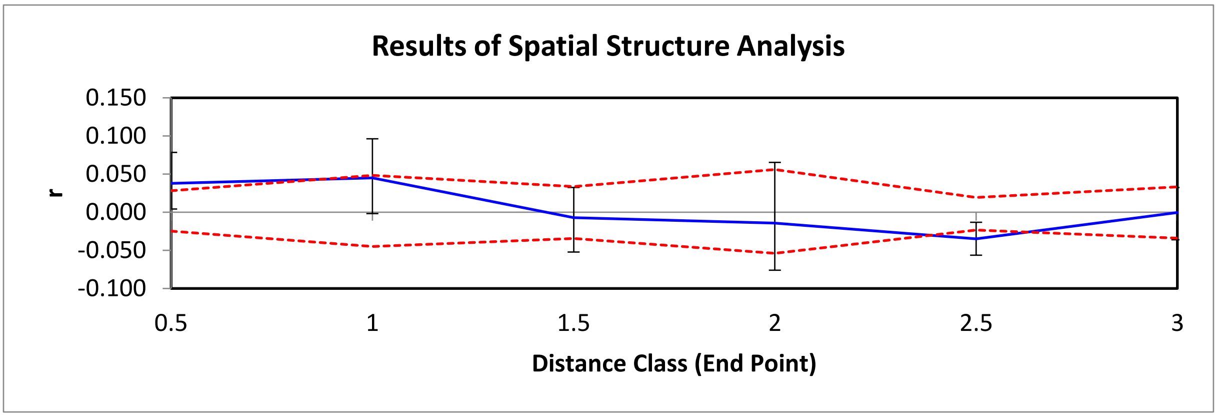

Figure S1.5. Local spatial autocorrelation (*r*) for even distance classes (0-3 km), for the spatial Population2 of *Q. petraea*, and their 95% confidence intervals (CI). The 95% CI around the null hypothesis of no genetic structure is shown with dashed lines based on 999 permutations; error bars bound the 95% confidence interval about *r* as determined by 1000 bootstrap resampling.

Isolation-by-distance

A Mantel test was performed to detect a relationship between genetic data (GD) and the spatial layout of sampling locations (GGD), Figure S1.6. Results regarding the IBD regression model are inconsistent: a rejection of the model was observed for the whole set of samples and the spatial Population1, whereas non-rejection for the spatial Population2 (Rxy =0.226, *P*=0.01; Rxy 0.247, *P*=0.01) based on original and transformed data, respectively).

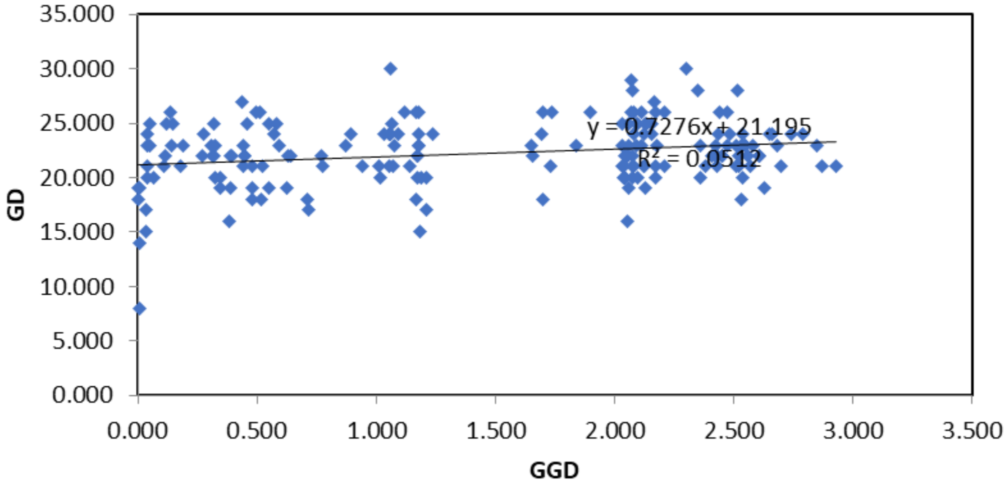

Figure S1.6. Relationship between pairwise genetic distance (GD) based on 12 SSR loci and geographic distances (GGD) for a set of 20 oak trees *Q. petraea* (spatial Population2) (Mantel test, *P*=0.01). GGD in km.

Analysis of DNA content by flow cytometry

To assess the ploidy level of cells, an internal standard was used to compare the positions of the G1 peak in profiles from different genotypes of oaks. The results of DNA content of mixed samples (*Quercus petraea.* and *Raphanus sativus*) are shown in Figure S1.7. The oak and radish formed narrow and high DNA peaks indicating a good resolution of DNA content measurements. The oak DNA peak was generally lower than that of the radish peak. Figure S1.7.

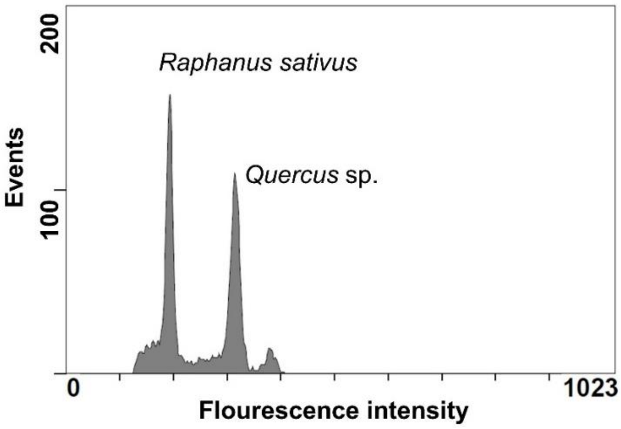

Figure S1.7. Example of a flow cytometric relative fluorescence intensity (representing the relative DNA content) of propidium iodide-stained nuclei isolated from the *Quercus petraea* tree and – *Raphanus sativus* "Saxa. The samples showed peaks corresponding to the G0/G1 phase of the cell cycle (non-replicated nuclear status); fluorescence intensity is expressed as channel numbers.

The determination of the nuclear DNA content of 48 accessions (each multiplied by three to eight biological replications, after excluding outliers) of *Q. petraea* ranged from 1.793pg to 1.900pg. The

mean value is equal to 1.848±SD 0.0285pg and the mean value of CV 0.5% for the whole data set, whereas for individual tree CV ranged from 0.00 to 1.037%, Table S1.6.

Since the equality of variance for the analyzed data set is violated, the Kruskal-Wallis test was employed to examine differences in 2C-DNA amounts among the trees. The value of H = 284.329, *P*=0.000, N=332 was tested against the chi-square distribution chi-square (χ^2) = 240.638, df 48, *P*=0.000. The tests suggest significant differences among analyzed trees in DNA contents (H>χ^2), *P*=0.000.

Table S1.6. Summary statistics for measurements of 2CDNA content of *Q. petraea* (2C - diplophasic genome size).

ID	N	Mean	Min	Max	SD	CV (%)
1	8	1.894	1.88	1.92	0.013	0.688
2	8	1.874	1.86	1.9	0.015	0.804
3	7	1.834	1.83	1.84	0.005	0.291
4	7	1.873	1.85	1.89	0.017	0.910
5	8	1.858	1.85	1.87	0.007	0.381
6	7	1.854	1.85	1.86	0.005	0.288
11	8	1.826	1.82	1.84	0.007	0.407
12	8	1.878	1.87	1.9	0.010	0.551
13	8	1.841	1.83	1.87	0.015	0.792
14	6	1.850	1.84	1.86	0.009	0.483
15	7	1.831	1.82	1.85	0.011	0.584
17	6	1.875	1.86	1.89	0.010	0.559
22	8	1.851	1.84	1.87	0.011	0.608
23	8	1.900	1.88	1.92	0.012	0.629
24	8	1.825	1.81	1.84	0.011	0.586
25	8	1.841	1.83	1.86	0.011	0.612
26	7	1.806	1.79	1.82	0.011	0.628
27	6	1.865	1.85	1.88	0.014	0.739
28	7	1.850	1.84	1.86	0.008	0.441
29	6	1.850	1.85	1.85	0.000	0.000
30	6	1.865	1.86	1.87	0.005	0.294
31	5	1.866	1.85	1.88	0.015	0.813
32	6	1.830	1.81	1.85	0.019	1.037
33	6	1.835	1.82	1.84	0.008	0.456
34	8	1.818	1.80	1.83	0.010	0.570
35	7	1.793	1.78	1.81	0.014	0.770
36	7	1.839	1.83	1.85	0.009	0.489
37	7	1.841	1.83	1.85	0.009	0.489
38	8	1.848	1.84	1.87	0.010	0.560
39	7	1.866	1.86	1.87	0.005	0.286
40	8	1.828	1.82	1.85	0.010	0.566
41	8	1.798	1.78	1.81	0.010	0.576
42	7	1.824	1.82	1.83	0.005	0.293
43	8	1.846	1.83	1.86	0.011	0.574

ID	N	Mean	Min	Max	SD	CV (%)
44	6	1.845	1.84	1.85	0.005	0.297
45	8	1.871	1.86	1.9	0.014	0.725
30B	5	1.836	1.83	1.84	0.005	0.298
34F	8	1.860	1.85	1.87	0.009	0.498
37A	6	1.853	1.85	1.86	0.005	0.279
37G	3	1.840	1.84	1.84	0.000	0.000
38F	5	1.836	1.83	1.84	0.005	0.298
42+	8	1.876	1.87	1.89	0.007	0.397
42B	5	1.834	1.83	1.84	0.005	0.299
42F	8	1.829	1.82	1.84	0.008	0.456
45+	3	1.830	1.83	1.83	0.000	0.000
45A	5	1.866	1.86	1.87	0.005	0.294
45B	7	1.876	1.87	1.89	0.008	0.419
45C	7	1.861	1.84	1.88	0.016	0.845
**Total**	**332**	**1.848**	**1.78**	**1.92**	**0.025**	**1.369**

Discussion

We studied patterns of (cyto)genetic interspecific and intraspecific variation in oaks originated from two stands in Poland using a set of highly polymorphic and co-dominantly inherited microsatellite loci, either linked to genes of adaptive importance or neutral. The cytogenetic diversity was estimated using propidium iodide flow cytometry. These cytogenetic background data are valuable for the subsequent taxonomic and ecological studies.

Tree identification based on molecular data

To test the morphological species hypothesis, we used the NJ tree based on Provesti`s distances from the SSR data to expect the genotypes to separate into two main clusters corresponding to *Q. robur* and *Q. petraea*. The bootstrap values at internal nodes in this tree indicated the phylogenetic relationships of both species are not well resolved by this method. However, genetic differentiation between both species as measured by F_{ST} value is low but significant. In this respect, our results are congruent with those obtained by Muir and Schloetterer [19]; F_{ST} = 0.058, P<0.001, and F_{ST} = 0.050; P<0.0001 in our study and Muir and Schloetterer [19], respectively. Our results are also consistent with allozyme, RAPD, and cpDNA marker analysis, which showed that the genetic differentiation between the species is very low [81,82]. We find no evidence that using only three species-specific SSR markers as recommended by Neophytou et al. [32] is sufficient to discriminate between both species; thus, there is the need for an in-depth genetic analysis of the analyzed oaks.

Diversity and structure of pooled population of Q. petraea

A short-distance spatial clustering of genotypes is theoretically expected if restricted seed and/or pollen dispersal is more frequent than long-distance dispersal [83]. Moreover, if local dispersion dominates, then the resulting pattern of neutral genetic variation should be consistent with the isolation by distance (IBD) hypothesis [72,84], see also [85,86]. Populations with relatively high dispersal have little structure and low autocorrelations [87]. An inconsistent picture emerged for the two studied spatial oak populations. Varied tree density within the populations may affect the analysis because the Mantel test and spatial autocorrelation tests used in this study do not distinguish between patterns resulting from tree clustering from those resulting from isolation by distance [87-90]. For example, as shown by [88], clumping increased the parameter Moran's I (closely related to autocorrelation coefficient r analyzed in this study). Moreover, the number of sampled individuals may bias the results, see [87]. We also cannot exclude such possibilities that the IBD model and the spatial autocorrelation analysis do not account for the spread of oak trees at the spatial scale we studied.

The obtained results of the SSR analyses, such as small, although significant genetic differentiation between spatial groups of oaks, no distance effect (no isolation by distance) for the total sample, no linkage disequilibrium between loci ($rbar_d$ close to 0), high genetic variation are consistent with the prevalence of extensive gene flow (presumably pollen-mediated). Nevertheless, we observe genetic diversity within the analyzed population of *Q. petraea*, as shown by the NJ tree. Other processes may explain such a nonspatial pattern of genetic diversity. For example, we cannot exclude that re-afforestation using mixed seed lots may alter the amount and distribution of genetic variation. Also, phenological and recruitment dynamics can determine such nonspatial clustering of genetic variation [91,92].

In our study, the average genome size in *Q. petraea* is 1.848±SD 0.0285pg and is generally considered small compared to other angiosperm plants [93,94]. All individuals of *Q. petraea* present only one cytotype assumed to be the most common ploidy level found in the literature, diploidy (our estimations are 24.27% lower than in triploids determined by Dzialuk et al. [38]). Also, our results show that neutral molecular variation using ten SSR primer pairs gave typical banding patterns as expected in diploid species. This means that none of the used primer pairs showed more than two bands per locus per genotype, see also [38]. The result may support the view that genome size and ploidy level are conserved in the analyzed oaks, see also [95].

Nevertheless, based on previous research, 2CDNA levels estimated for the "pure diploid species *Q. petraea* and *Q. robur* vary significantly between studies [38,41-44]. Nuclear DNA content (2C)

obtained in this study is very similar to the values reported by Greilhuber [42] for *Q. petraea* (2C DNA content = 1.79pg) and Favre and Brown [43]– 2C DNA contents 1.84±SD 0.01 and 1.87 ±SD 0.02 for *Q. robur and Q. petraea,* respectively. Nevertheless, higher than that reported by [44] (2C DNA = 1.58 pg), which could be consequences a methodology used, for example, sampling, fixation, preparation, and staining [45].

Previous genome size estimations within *Q. petraea* using the cytometry technique revealed nuclear DNA content heterogeneity, as shown in our study by the Kruskal-Wallis test. Moreover, the difference between the mean 2C DNA content obtained in this study is 12.34% higher than that determined by Dzialuk et al. [38] for diploids. Several reasons may be responsible for the results, such as chromosomal variations (e.g., aneuploidy, presence of B-chromosomes) or differences in the content of repetitive DNA or/and technique-related variations [96]. For example, the number of leaves analyzed can affect the results, especially when more than one leaf is analyzed per plant, as in our work. There is no formal consensus on the acceptable error in estimating the DNA content (the proper maximum CV value of the average DNA content in a population of cells G0/G1). However, a CV threshold of 6% is accepted in most studies, and such measurements are considered correctly performed [97]. In our study, the maximum value of the coefficient of variation for a tree is just over 1%, indicating that the measurements were correctly made. Nevertheless, despite efforts to select leaves of the same vital parameters (e.g., age and physiological condition) small differences in these variables may correlate with the content of secondary metabolites that, in turn, may slightly influence the staining, see also [98-101], hence it was necessary to reject the outliers. Repeated measurements of plants with known chromosome numbers should resolve whether differences between trees are due to karyological instability, such as aneuploidy or the presence of B-chromosomes [45], much deeper research is needed to show that these differences are due to duplicated or deleted DNA regions [102].

References

1. Aas, G. Taxonomical impact of morphological variation in *Quercus robur* and *Q. petraea*: a contribution to the hybrid controversy. *In Proceedings of the Annales des Sciences Forestières*, **1993**; pp. 107s-113s.
2. Eaton, E.; Caudullo, G.; Oliveira, S.; De Rigo, D. *Quercus robur* and *Quercus petraea* in Europe: distribution, habitat, usage and threats. *European atlas of forest tree species* **2016**, 160-163.
3. Fairbairn, W. Preliminary light-intensity study under sessile and pedunculate oak. *Forestry: An International Journal of Forest Research* **1954**, *27*, 1-6.
4. McShea, W.J.; Healy, W.M.; Devers, P.; Fearer, T.; Koch, F.H.; Stauffer, D.; Waldon, J. Forestry matters: decline of oaks will impact wildlife in hardwood forests. *The Journal of Wildlife Management* **2007**, *71*, 1717-1728.
5. Wetherbee, R.; Birkemoe, T.; Skarpaas, O.; Sverdrup-Thygeson, A. Hollow oaks and beetle functional diversity: Significance of surroundings extends beyond taxonomy. *Ecology and evolution* **2020**, *10*, 819-831.

6. Streiff, R.; Ducousso, A.; Lexer, C.; Steinkellner, H.; Gloessl, J.; Kremer, A. Pollen dispersal inferred from paternity analysis in a mixed oak stand of *Quercus robur* L. and *Q. petraea* (Matt.) Liebl. *Molecular Ecology* **1999**, *8*, 831-841.
7. Chybicki, I.; Burczyk, J. Realized gene flow within mixed stands of *Quercus robur* L. and *Q. petraea* (Matt.) L. revealed at the stage of naturally established seedling. *Molecular Ecology* **2010**, *19*, 2137-2151.
8. Kremer, A.; Kleinschmit, J.; Cottrell, J.; Cundall, E.P.; Deans, J.D.; Ducousso, A.; König, A.O.; Lowe, A.J.; Munro, R.C.; Petit, R.J. Is there a correlation between chloroplastic and nuclear divergence, or what are the roles of history and selection on genetic diversity in European oaks? *Forest Ecology and Management* **2002**, *156*, 75-87.
9. Lexer, C.; Kremer, A.; Petit, R. Shared alleles in sympatric oaks: recurrent gene flow is a more parsimonious explanation than ancestral polymorphism. *Molecular Ecology* **2006**, *15*, 2007-2012.
10. Stanley, R.G.; Linskens, H.F. *Pollen: Biology Biochemistry Management*; Springer: New York, **2012**.
11. Moran, E.V.; Willis, J.; Clark, J.S. Genetic evidence for hybridization in red oaks (*Quercus* sect. Lobatae, Fagaceae). *American Journal of Botany* **2012**, *99*, 92-100.
12. Di Pietro, R.; Di Marzio, P.; Antonecchia, G.; Conte, A.L.; Fortini, P. Preliminary characterization of the *Quercus pubescens* complex in southern Italy using molecular markers. *Acta Botanica Croatica* **2020**, *79*, 0-0.
13. Krutovsky, K.V.; Burczyk, J.; Chybicki, I.; Finkeldey, R.; Pyhäjärvi, T.; Robledo-Arnuncio, J.J. Gene flow, spatial structure, local adaptation, and assisted migration in trees. In *Genomics of tree crops*, Schnell, R., Priyadarshan, P., Eds.; Springer: New York, **2012**; pp. 71-116.
14. Browne, L.; Wright, J.W.; Fitz-Gibbon, S.; Gugger, P.F.; Sork, V.L. Adaptational lag to temperature in valley oak (*Quercus lobata*) can be mitigated by genome-informed assisted gene flow. *Proceedings of the National Academy of Sciences* **2019**, *116*, 25179-25185.
15. Petit, R.J.; Wagner, D.B.; Kremer, A. Ribosomal DNA and chloroplast DNA polymorphisms in a mixed stand of *Quercus robur* and *Q. petraea*. *Annales Des Sciences Forestières* **1993**, *50*, 41–47.
16. Bacilieri, R.; Ducousso, A.; Kremer, A. Genetic, morphological, ecological and phenological differentiation between *Quercus petraea* (Matt.) Liebl. and *Quercus robur* L. in a mixed stand of northwest of France. *Silvae genetica* **1995**, *44*, 1-9.
17. Coart, E.; Lamote, V.; De Loose, M.; Van Bockstaele, E.; Lootens, P.; Roldan-Ruiz, I. AFLP markers demonstrate local genetic differentiation between two indigenous oak species [*Quercus robur* L. and *Quercus petraea* (Matt.) Liebl.] in Flemish populations. *Theoretical and Applied Genetics* **2002**, *105*, 431-439.
18. Scotti-Saintagne, C.; Mariette, S.; Porth, I.; Goicoechea, P.G.; Barreneche, T.; Bodénes, C.; Burg, K.; Kremer, A. Genome scanning for interspecific differentiation between two closely related oak species [*Quercus robur* L. and *Q. petraea* (Matt.) Liebl.]. *Genetics* **2004**, *168*, 1615-1626.
19. Muir, G.; Schloetterer, C. Evidence for shared ancestral polymorphism rather than recurrent gene flow at microsatellite loci differentiating two hybridizing oaks (*Quercus* spp.). *Molecular ecology* **2005**, *14*, 549-561.
20. Bauer, N.; Horvat, T.; Biruš, I.; Vičić, V.; Zoldoš, V. Nucleotide sequence, structural organization and length heterogeneity of ribosomal DNA intergenic spacer in *Quercus petraea* (Matt.) Liebl. and *Q. robur* L. *Molecular Genetics and Genomics* **2009**, *281*, 207-221.
21. Leroy, T.; Rougemont, Q.; Dupouey, J.L.; Bodénès, C.; Lalanne, C.; Belser, C.; Labadie, K.; Le Provost, G.; Aury, J.M.; Kremer, A. Massive postglacial gene flow between European white oaks uncovered genes underlying species barriers. *New Phytologist* **2020**, *226*, 1183-1197.
22. Cannon, C.H.; Scher, C.L. Exploring the potential of gametic reconstruction of parental genotypes by F1 hybrids as a bridge for rapid introgression. *Genome* **2017**, *60*, 713-719.
23. Gardiner, A. Pedunculate and sessile oak (*Quercus robur* l. and *Quercus petraea* (mattuschka) liebl.) a review of the hybrid controversy. *Forestry* **1970**, *43*, 151-160.

24. Rushton, B. Natural hybridization within the genus *Quercus* L. In Proceedings of the Annales des Sciences Forestières, **1993**; pp. 73-90.
25. Alexandre, H.; Truffaut, L.; Ducousso, A.; Louvet, J.-M.; Nepveu, G.; Torres-Ruiz, J.M.; Lagane, F.; Firmat, C.; Musch, B.; Delzon, S. *In situ* estimation of genetic variation of functional and ecological traits in *Quercus petraea* and *Q. robur*. *Tree genetics & genomes* **2020**, *16*, 1-23.
26. Proietti, E.; Filesi, L.; Di Marzio, P.; Di Pietro, R.; Masin, R.; Conte, A.L.; Fortini, P. Morphology, geometric morphometrics, and taxonomy in relict deciduous oaks woods in northern Italy. *Rendiconti Lincei. Scienze Fisiche e Naturali* **2021**, *32*, 549-564.
27. Muller-Starck, G.; Ziehe, M. Genetic Variation in Populations of *Fagus sylvatica* L., *Quercus robur* L., and *Quercus petraea* (Matt.) Liebl. In *Genetic Variation in Europaean Populations of Forest Trees*, Muller-Starck, G., Ziehe, M., Eds.; Sauerlander's Verlag: Frankfurt am Main, Germany, **1991**; p. 125.
28. Gömöry, D.; Yakovlev, I.; Zhelev, P.; Jedináková, J.; Paule, L. Genetic differentiation of oak populations within the *Quercus robur/Quercus petraea* complex in Central and Eastern Europe. *Heredity* **2001**, *86*, 557-563.
29. Siegismund, H.R.; Jensen, J.S. Intrapopulation and interpopulation genetic variation of *Quercus* in Denmark. *Scandinavian Journal of Forest Research* **2001**, *16*, 103-116.
30. Hipp, A.L.; Manos, P.S.; Hahn, M.; Avishai, M.; Bodénès, C.; Cavender-Bares, J.; Crowl, A.A.; Deng, M.; Denk, T.; Fitz-Gibbon, S. Genomic landscape of the global oak phylogeny. *New Phytologist* **2020**, *226*, 1198-1212.
31. Jurkšienė, G.; Baranov, O.Y.; Kagan, D.I.; Kovalevič-Razumova, O.A.; Baliuckas, V. Genetic diversity and differentiation of pedunculate (*Quercus robur*) and sessile (*Q. petraea*) oaks. *Journal of Forestry Research* **2020**, *31*, 2445-2452.
32. Neophytou, C.; Aravanopoulos, F.A.; Fink, S.; Dounavi, A. Detecting interspecific and geographic differentiation patterns in two interfertile oak species (*Quercus petraea* (Matt.) Liebl. and *Q. robur* L.) using small sets of microsatellite markers. *Forest Ecology and Management* **2010**, *259*, 2026-2035.
33. Moore, D.M. *Flora Europaea check-list and chromosome index*; Cambridge University Press: Cambridge, **1982**; Volume 1.
34. Cousens, J. Variation of some diagnostic characters of the sessile and pedunculate oaks and their hybrids in Scotland. *Watsonia* **1963**, *5*, 273-286.
35. Burda, R.; Shchepotiev, F. Spontaneous polyploidy in seedlings of multi-seeded acorns of *Quercus robur* L. *Cytology and Genetics* **1973**, *7*, 140-143.
36. Butorina, A. Cytogenetic study of diploid and spontaneous triploid oaks, *Quercus robur* L. In Proceedings of the Annales des Sciences Forestières, **1993**; pp. 144-150.
37. Naujoks, G.; Hertel, H.; Ewald, D. Characterization and propagation of an adult triploid pedunculate oak (*Quercus robur* L.). *Silvae Genetica* **1995**, *44*, 282-285.
38. Dzialuk, A.; Chybicki, I.; Welc, M.; Śliwińska, E.; Burczyk, J. Presence of triploids among oak species. *Annals of Botany* **2007**, *99*, 959-964.
39. Burgarella, C.; Lorenzo, Z.; Jabbour-Zahab, R.; Lumaret, R.; Guichoux, E.; Petit, R.; Soto, A.; Gil, L. Detection of hybrids in nature: application to oaks (*Quercus suber* and *Q. ilex*). *Heredity* **2009**, *102*, 442-452.
40. Viscosi, V.; Antonecchia, G.; Lepais, O.; Fortini, P.; Gerber, S.; Loy, A. Leaf shape and size differentiation in white oaks: assessment of allometric relationships among three sympatric species and their hybrids. *International Journal of Plant Sciences* **2012**, *173*, 875-884.
41. Olszewska, M.J.; Osiecka, R. The relationship between 2 C DNA content, systematic position, and the level of nuclear DNA endoreplication during differentiation of root parenchyma in some dicotyledonous shrubs and trees. comparison with Herbaceous species. *Biochemie und Physiologie der Pflanzen* **1984**, *179*, 641-657.

42. Greilhuber, J. "Self-tanning", a new and important source of stoichiometric error in cytophotometric determination of nuclear DNA content in plants. *Plant Systematics and Evolution* **1988**, *158*, 87-96.
43. Favre, J.; Brown, S. A flow cytometric evaluation of the nuclear DNA content and GC percent in genomes of European oak species. In Proceedings of the Annales des sciences forestières, **1996**; pp. 915-917.
44. Ohri, D.; Ahuja, M. Giemsa C-banded karyotype in *Quercus* L. (oak). *Silvae Genetica* **1990**, *39*, 216-219.
45. Zoldoš, V.; Papeš, D.; Brown, S.; Panaud, O.; Siljak-Yakovlev, S. Genome size and base composition of seven *Quercus* species: inter-and intra-population variation. *Genome* **1998**, *41*, 162-168.
46. López-Aljorna, A.; Bueno, M.Á.; Aguinagalde, I.; Martín, J.P. Fingerprinting and genetic variability in cork oak (*Quercus suber* L.) elite trees using ISSR and SSR markers. *Annals of Forest Science* **2007**, *64*, 773-779.
47. Vieira, M.L.C.; Santini, L.; Diniz, A.L.; Munhoz, C.d.F. Microsatellite markers: what they mean and why they are so useful. *Genetics and molecular biology* **2016**, *39*, 312-328.
48. Lefort, F.; Lally, M.; Thompson, D.; Douglas, G. Morphological traits, microsatellite fingerprinting and genetic relatedness of a stand of elite oaks (*Q. robur* L.) at Tullynally, Ireland. *Silvae Genetica* **1998**, *47*, 257-261.
49. Loureiro, J.; Pinto, G.; Lopes, T.; Doležel, J.; Santos, C. Assessment of ploidy stability of the somatic embryogenesis process in *Quercus suber* L. using flow cytometry. *Planta* **2005**, *221*, 815-822.
50. Kampfer, S.; Lexer, C.; Glossl, J.; Steinkellner, H. Characterization of (GA) n microsatellite loci from *Quercus robur*. *Hereditas* **1998**, *129*, 1-86.
51. Steinkellner, H.; Lexer, C.; Turetschek, E.; Glössl, J. Conservation of (GA) n microsatellite loci between *Quercus* species. *Molecular Ecology* **1997**, *6*, 1189-1194.
52. Prevosti, A.; Ocana, J.; Alonso, G. Distances between populations of *Drosophila subobscura*, based on chromosome arrangement frequencies. *Theoretical and Applied Genetics* **1975**, *45*, 231-241.
53. Kamvar, Z.N.; Tabima, J.F.; Grünwald, N.J. Poppr: an R package for genetic analysis of populations with clonal, partially clonal, and/or sexual reproduction. *PeerJ* **2014**, *2*, e281.
54. Peakall, R.; Smouse, P.E. GENALEX 6: genetic analysis in Excel. Population genetic software for teaching and research. *Molecular ecology notes* **2006**, *6*, 288-295.
55. Liu, P.; Que, Y.; Pan, Y.-B. Highly polymorphic microsatellite DNA markers for sugarcane germplasm evaluation and variety identity testing. *Sugar Tech* **2011**, *13*, 129-136.
56. Wattier, R.; Engel, C.; Saumitou-Laprade, P.; Valero, M. Short allele dominance as a source of heterozygote deficiency at microsatellite loci: experimental evidence at the dinucleotide locus Gv1CT in *Gracilaria gracilis* (Rhodophyta). *Molecular ecology* **1998**, *7*, 1569-1573.
57. Chapuis, M.-P.; Estoup, A. Microsatellite null alleles and estimation of population differentiation. *Molecular biology and evolution* **2007**, *24*, 621-631.
58. Adamack, A.T.; Gruber, B. PopGenReport: simplifying basic population genetic analyses in R. *Methods in Ecology and Evolution* **2014**, *5*, 384-387.
59. R_Core_Team *R: A language and environment for statistical computing*, R Foundation for Statistical Computing: Vienna, Austria, **2021**.
60. Chakraborty, R.; Andrade, M.d.; Daiger, S.; Budowle, B. Apparent heterozygote deficiencies observed in DNA typing data and their implications in forensic applications. *Annals of Human Genetics* **1992**, *56*, 45-57.
61. Brookfield, J. A simple new method for estimating null allele frequency from heterozygote deficiency. *Molecular ecology* **1996**, *5*, 453-455.
62. Di Fonzo, M.M.; Pelletier, F.; Clutton-Brock, T.; Pemberton, J.M.; Coulson, T. The population growth consequences of variation in individual heterozygosity. *PLoS One* **2011**, *6*, e19667.

63. Coulon, A. GENHET: an easy-to-use R function to estimate individual heterozygosity. *Molecular Ecology Resources* **2010**, *10*, 167-169.
64. Wang, J.; Shete, S. Testing departure from hardy–Weinberg proportions. In *Statistical Human Genetics. Methods in Molecular Biology (Methods and Protocols)*, Elston, R., Satagopan, J., Sun, S., Eds.; Humana Press: Totowa, USA, **2012**; Volume 850, pp. 77-102.
65. Paradis, E. pegas: an R package for population genetics with an integrated–modular approach. *Bioinformatics* **2010**, *26*, 419-420.
66. Jombart, T. adegenet: a R package for the multivariate analysis of genetic markers. *Bioinformatics* **2008**, *24*, 1403-1405.
67. Waples, R.S.; Gaggiotti, O. What is a population? An empirical evaluation of some genetic methods for identifying the number of gene pools and their degree of connectivity. *Molecular ecology* **2006**, *15*, 1419-1439.
68. Chen, K.Y.; Marschall, E.A.; Sovic, M.G.; Fries, A.C.; Gibbs, H.L.; Ludsin, S.A. assign POP: An r package for population assignment using genetic, non-genetic, or integrated data in a machine-learning framework. *Methods in Ecology and Evolution* **2018**, *9*, 439-446.
69. Anderson, E. Assessing the power of informative subsets of loci for population assignment: standard methods are upwardly biased. *Molecular ecology resources* **2010**, *10*, 701-710.
70. Waples, R.S. High-grading bias: subtle problems with assessing power of selected subsets of loci for population assignment. *Molecular Ecology* **2010**, *19*, 2599-2601, doi:https://doi.org/10.1111/j.1365-294X.2010.04675.x.
71. Villanueva, R.A.M.; Chen, Z.J. *ggplot2: elegant graphics for data analysis*, Taylor & Francis: 2019.
72. Wright, S. Isolation by distance. *Genetics* **1943**, *28*, 114.
73. Milgroom, M.G. Recombination and the multilocus structure of fungal populations. *Annual review of phytopathology* **1996**, *34*, 457-477.
74. Nagylaki, T. The evolution of one-and two-locus systems. *Genetics* **1976**, *83*, 583-600.
75. Garnier-Géré, P.; Chikhi, L. Population subdivision, Hardy–Weinberg equilibrium and the Wahlund effect. *eLS* **2013**.
76. Galbraith, D.W.; Harkins, K.R.; Maddox, J.M.; Ayres, N.M.; Sharma, D.P.; Firoozabady, E. Rapid flow cytometric analysis of the cell cycle in intact plant tissues. *Science* **1983**, *220*, 1049-1051.
77. Greilhuber, J. Intraspecific variation in genome size in angiosperms: identifying its existence. *Annals of Botany* **2005**, *95*, 91-98.
78. Komsta, L. *Package 'outliers'*, Medical University of Lublin: Lublin, **2011**.
79. Paetkau, D. The molecular basis and evolutionary history of a microsatellite null allele in bears. *Molecular ecology* **1995**, *4*, 519-520.
80. Paetkau, D.; Waits, L.P.; Clarkson, P.L.; Craighead, L.; Strobeck, C. An empirical evaluation of genetic distance statistics using microsatellite data from bear (Ursidae) populations. *Genetics* **1997**, *147*, 1943-1957.
81. Barreneche, T.; Bahrman, N.; Kremer, A. Two dimensional gel electrophoresis confirms the low level of genetic differentiation between *Quercus robur* L. and *Quercus petraea* (Matt.) Liebl. *International Journal of Forest Genetics* **1996**, *3*, 89-92.
82. Bodénès, C.; Labbé, T.; Pradère, S.; Kremer, A. General vs. local differentiation between two closely related white oak species. *Molecular Ecology* **1997**, *6*, 713-724.
83. Jordano, P. What is long-distance dispersal? And a taxonomy of dispersal events. *Journal of Ecology* **2017**, *105*, 75-84.
84. Wright, S. The genetical structure of populations. *Annals of Eugenics* **1949**, *15*, 323-354.
85. Zanetto, A.; Kremer, A. Geographical structure of gene diversity in *Quercus petraea* (Matt.) Liebl. I. Monolocus patterns of variation. *Heredity* **1995**, *75*, 506-517.
86. Mariette, S.; Cottrell, J.; Csaikl, U.M.; Goikoechea, P.; Konig, A.; Lowe, A.J.; Van Dam, B.C.; Barreneche, T.; Bodénès, C.; Streiff, R. Comparison of levels of genetic diversity detected with

AFLP and microsatellite markers within and among mixed *Q. petraea* (Matt.) Liebl. and *Q. robur* L. stands. *Silvae genetica* **2002**, *51*, 72-79.
87. Epperson, B. Estimating dispersal from short distance spatial autocorrelation. *Heredity* **2005**, *95*, 7-15.
88. Doligez, A.; Baril, C.; Joly, H.I. Fine-scale spatial genetic structure with nonuniform distribution of individuals. *Genetics* **1998**, *148*, 905-919.
89. Chung, M.Y.; Epperson, B.K.; Gi Chung, M. Genetic structure of age classes in *Camellia japonica* (Theaceae). *Evolution* **2003**, *57*, 62-73.
90. Meirmans, P.G.; Goudet, J.; Consortium, I.; GAGGIOTTI, O.E. Ecology and life history affect different aspects of the population structure of 27 high-alpine plants. *Molecular Ecology* **2011**, *20*, 3144-3155.
91. Slavov, G.; Leonardi, S.; Adams, W.; Strauss, S.; DiFazio, S. Population substructure in continuous and fragmented stands of *Populus trichocarpa*. *Heredity* **2010**, *105*, 348-357.
92. Piotti, A.; Leonardi, S.; Heuertz, M.; Buiteveld, J.; Geburek, T.; Gerber, S.; Kramer, K.; Vettori, C.; Vendramin, G.G. Within-population genetic structure in beech (*Fagus sylvatica* L.) stands characterized by different disturbance histories: does forest management simplify population substructure? *PLoS One* **2013**, *8*, e73391.
93. Leitch, I.; Johnston, E.; Pellicer, J.; Hidalgo, O.; Bennett, M. Angiosperm DNA C-values database. **2019**.
94. Wei, G.; Li, X.; Fang, Y. Sympatric genome size variation and hybridization of four oak species as determined by flow cytometry genome size variation and hybridization. *Ecology and evolution* **2021**, *11*, 1729-1740.
95. Chen, S.-C.; Cannon, C.H.; Kua, C.-S.; Liu, J.-J.; Galbraith, D.W. Genome size variation in the Fagaceae and its implications for trees. *Tree genetics & genomes* **2014**, *10*, 977-988.
96. Šmarda, P.; Bureš, P. Understanding intraspecific variation in genome size in plants. *Preslia* **2010**, *82*, 41-61.
97. Darzynkiewicz, Z.; Huang, X.; Zhao, H. Analysis of cellular DNA content by flow cytometry. *Current protocols in immunology* **2017**, *119*, 1-20.
98. Scalbert, A.; Haslam, E. Polyphenols and chemical defence of the leaves of *Quercus robur*. *Phytochemistry* **1987**, *26*, 3191-3195.
99. Fernández de Simón, B.; Cadahía, E.; Conde, E.; García-Vallejo, M.C. Low molecular weight phenolic compounds in Spanish oak woods. *Journal of Agricultural and Food Chemistry* **1996**, *44*, 1507-1511.
100. Greilhuber, J. Intraspecific variation in genome size: a critical reassessment. *Annals of Botany* **1998**, *82*, 27-35.
101. Luczaj, L.; Adamczak, A.; Duda, M. Tannin content in acorns (*Quercus* spp.) from Poland. *Dendrobiology* **2014**, *72*.
102. Cavallini, A.; Natali, L.; Giordani, T.; Durante, M.; Cionini, P. Nuclear DNA changes within *Helianthus annuus* L.: variations in the amount and methylation of repetitive DNA within homozygous progenies. *Theoretical and Applied Genetics* **1996**, *92*, 285-291.

Supplementary File S2

Details on the statistical results presented in Table 1.

We sampled caterpillars from oak trees and determined a range of potential predictors of their abundance, parasitism rates, Simpson Diversity, and functional traits (see main text). The caterpillar densities were square root transformed to approximate normally distributed residuals. We first calculated Spearman's correlation coefficients between all predictors and all response variables (Table S2.1). We subsequently took the "dredge" approach using the R Package MuMIn (Barton 2009). Thus, for each category, we fitted all possible Ordinary Least Squares (OLS) models with the predictors and their interactions with phylogenetic isolation, and we sorted them by a corrected by small sample sizes Akaike Information Criterium corrected for small sample sizes (AICc) value. Notably, AICc values tended to be similar among the top models, hence our decision to report the incidence of predictors in the top-ten models (see Table 1 in the main text). To complement Table 1, we below summarize the top-ten models for each dependent variable and provide specifics for the top model, including residual plots (Tables S2.2 – S2.27, Figures S2.1 – S2.13). Outliers were identified as laying outside Cook's distances in the residuals vs leverage plots or those laying far from the line in Q-Q plots. Finally, we here address whether the effect of parasitism on the abundance of the most common species could be attributed to a methodological artefact (Figure S2.14).

Correlations between response variables and predictors:

Table S2.1. Spearman's Correlation Coefficients, where . denotes p < 0.1, * p < 0.05, ** p < 0.01, *** p < 0.001. All models used 25 trees, except for Simpson diversity and insect traits where 2 trees were excluded due to lack of data. Day = Sampling day, BB = Date of 50% Budburst, Diam = Trunk diameter at breast height, Par = Parasitism rate, Fcyt = Genome size, IH = Standardized Individual Heterozygosity based on the mean observed heterozygosity, PI = Phylogenetic Isolation, sdPI = Phylogenetic Heterogeneity of the neighbourhood expressed as the Standard Deviation of Phylogenetic Isolation, P. specialists = The proportion of host-plant specialists, and P. Particular Spec. = The proportion of species that only feed on oak out of the dominant neighbouring tree species; oak, hornbeam, beech, and pine.

	Day	BB	Diam	Par	IH	Fcyt	PI	sdPI
All caterpillars	-0.37 .	0.06	-0.30	-0.31	0.12	-0.14	0.52**	0.06
Casebearers	-0.22	0.18	-0.25	-0.26	-0.08	-0.14	0.44*	-0.11
C. flavipennella	-0.33	-0.16	-0.52 **	-0.71***	0.11	-0.02	0.25	-0.08
C. lutipennella	-0.06	0.13	-0.44 *	-0.27	-0.49 *	-0.07	0.29	-0.30
Semi-concealed	-0.32	0.16	-0.25	-0.24	0.23	-0.20	0.56**	0.10
C. quercana	-0.02	0.18	-0.09	-0.30	-0.13	0.13	0.15	-0.03
Free living	-0.39 .	-0.06	-0.32	-0.36 .	0.20	-0.12	0.50*	0.13
Geometrids	-0.27	-0.36 .	-0.37 .	-0.34	0.05	-0.19	0.18	0.21
Parasitism rates	0.33	0.00	0.39		-0.09	-0.11	-0.16	0.25
Simpson diversity	0.12	-0.12	0.14	0.09	-0.36 .	0.30	-0.26	-0.58 **
Wingspan	-0.30	-0.08	-0.14	-0.25	0.19	-0.03	0.23	0.10
P. Specialists	-0.28	-0.13	-0.32	-0.13	0.20	-0.16	-0.06	-0.06
P. Particular Spec.	-0.15	-0.05	-0.36 .	-0.16	-0.14	-0.19	-0.01	0.04

Results of the dredge procedure for each response variable reported in Table 1.

All caterpillars:

Table S2.2 Summary results of the top-ten models for caterpillar abundance (sqrt(abundance/leaf mass)). All models used data from 25 trees, and were weighted by the leaf mass of the sample. Cluster = Zielonka or Kaminsko, Day = Sampling day, BB = Budburst date, Diam = Diameter at breast height (cm), Fcyt = 2C nuclear DNA content (pg), IH = Standardized Individual Heterozygosity based on the mean observed heterozygosity, PI = Phylogenetic Isolation (ma), sdPI = Phylogenetic Heterogeneity expressed as the Standard Deviation of Phylogenetic Isolation.

Intercept	Cluster	Day	BB	Diam	Par	IH	Fcyt	PI	sdPI	Day:PI	BB:PI	Diam:PI	Par:PI	IH:PI	Fcyt:PI	PI:sdPI	df	logLik	AICc	delta	weight
0.23								0.0011									3	28.25	-49.37	0.00	0.067
0.37		-0.0037						0.0009									4	29.30	-48.61	0.76	0.046
0.27					-0.0009			0.0010									4	29.25	-48.51	0.86	0.043
0.20					0.0011			0.0020					-2.98E-05				5	30.38	-47.60	1.77	0.027
0.33		-0.0042			0.0020			0.0021					-3.89E-05				6	31.84	-47.02	2.35	0.021
0.23								0.0011	-0.0002								4	28.39	-46.77	2.59	0.018
0.28				-0.0009				0.0010									4	28.35	-46.70	2.66	0.018
0.38		-0.0029			-0.0007			0.0009									5	29.91	-46.67	2.70	0.017
-0.24							0.2477	0.0011									4	28.32	-46.63	2.73	0.017
0.22	+							0.0011									4	28.27	-46.54	2.83	0.016

Table S2.3 Summary results of the top model for all caterpillars, with estimated R^2 = 31%. Removing outlier 11 (tree 24, which had an exceptionally high density of semi-concealed caterpillars) does not qualitatively change this result presented below.

	Estimate	s.e.	t	p
Intercept	0.225	0.025	9.013	<0.001
PI	0.001	0.000	3.252	0.004

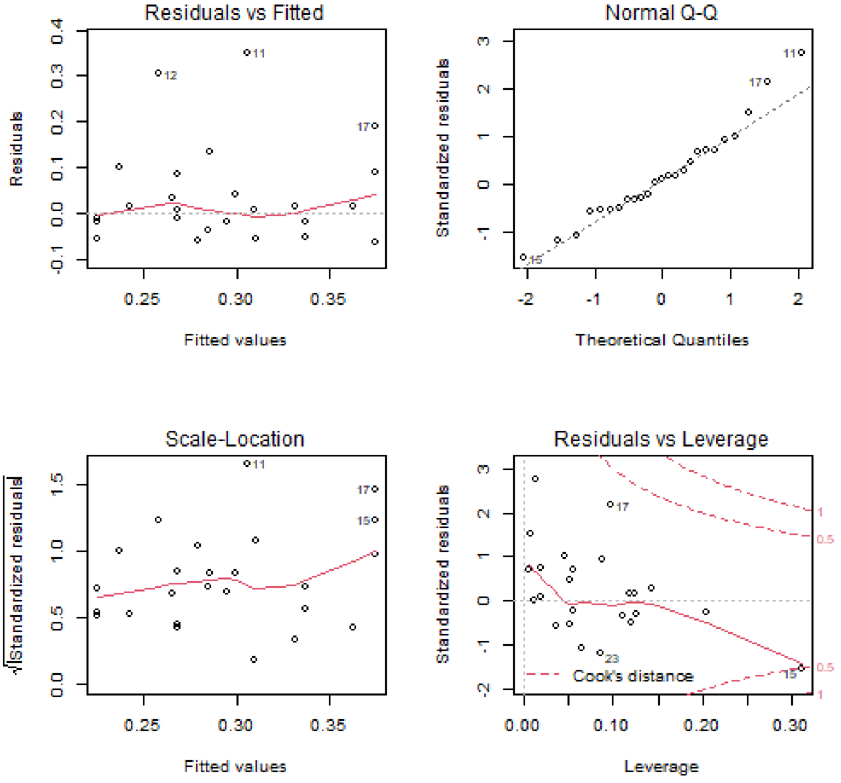

Figure S2.1 Plots of residuals of the top model for all caterpillars.

Casebearers:

Table S2.4. Top ten models for casebearer abundance. See Table S2.2 for abbreviations.

Intercept	Cluster	Day	BB	Diam	Par	IH	Fcyt	PI	sdPI	Day:PI	BB:PI	Diam:PI	Par:PI	IH:PI	Fcyt:PI	PI:sdPI	df	logLik	AICc	delta	weight
0.23					0.0016	-0.1447		0.0017					-3.39E-05				6	44.86	-73.04	0.00	0.063
0.41					0.0012	-0.3163		-0.0017					-2.95E-05	0.0033			7	46.71	-72.82	0.22	0.056
0.06	+				0.0015			0.0017					-3.21E-05				6	44.37	-72.07	0.98	0.038
0.08					0.0016			0.0017					-3.44E-05				5	42.29	-71.43	1.61	0.028
0.37	+				0.0011	-0.2891		-0.0020					-2.76E-05	0.0035			8	48.07	-71.15	1.90	0.024
0.56						-0.4133		-0.0042	-0.0006					0.0049			6	43.82	-70.98	2.06	0.022
0.18	+				0.0015	-0.1095		0.0017					-3.25E-05				7	45.75	-70.92	2.13	0.022
0.32		-0.0019			0.0020	-0.1795		0.0017					-3.78E-05				7	45.56	-70.54	2.51	0.018
0.52		-0.0020			0.0016	-0.3604		-0.0018					-3.35E-05	0.0034			8	47.69	-70.37	2.67	0.016
0.54					-0.0008	-0.3717		-0.0037						0.0043			6	43.20	-69.73	3.32	0.012

Table S2.5 Summary results of the top model for casebearers, with R^2 = 43%. When outlier 17 is removed (tree 31, with an exceptionally high density of casebearers), individual heterozygosity is significant at p = 0.018, while the effect of parasitism rate is not statistically significant (p=0.36).

	Estimate	s.e.	t	p
Intercept	0.315	0.081	3.863	0.001
IH	-0.149	0.078	-1.906	0.070
Par	-0.001	0.000	-1.697	0.105
PI	0.001	0.000	2.650	0.015

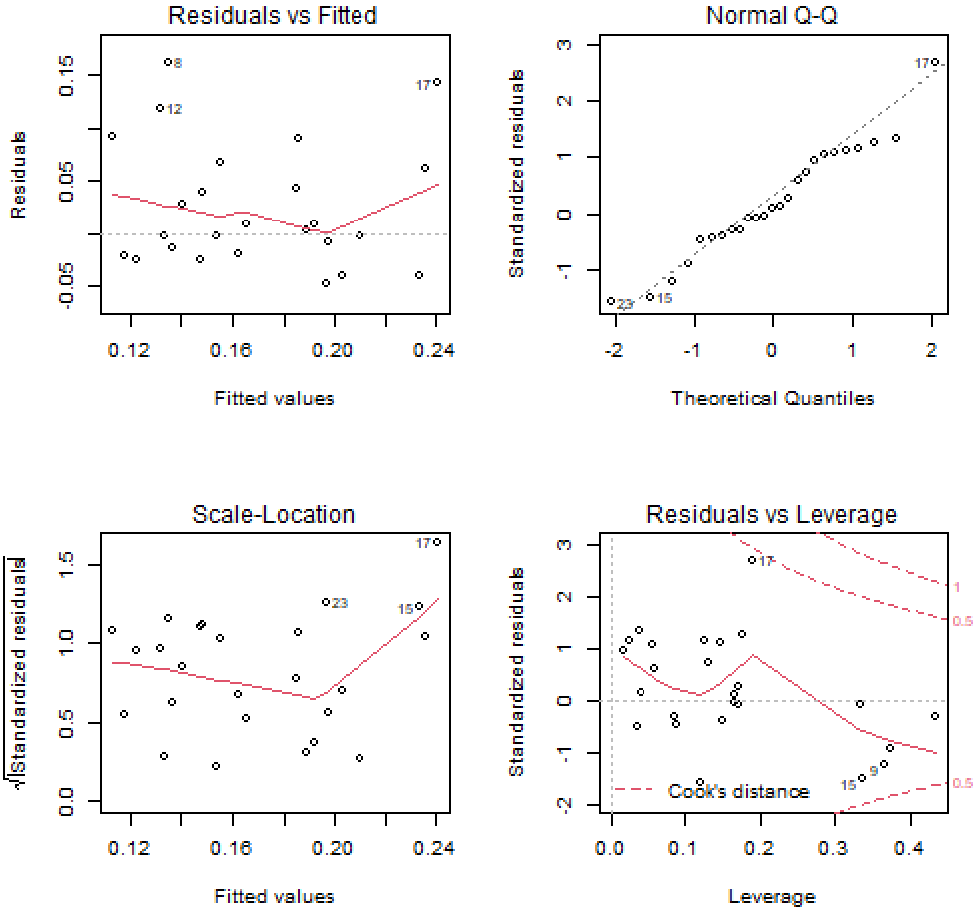

Figure S2.2 Plots of residuals of the top model for casebearers.

Coleophora flavipennella:

Table S2.6. Top ten models for *C. flavipennella* abundance. See Table S2.2 for abbreviations.

Intercept	Cluster	Day	BB	Diam	Par	IH	Fcyt	PI	sdPI	Day:PI	BB:PI	Diam:PI	Par:PI	IH:PI	Fcyt:PI	PI:sdPI	df	logLik	AICc	delta	weight
0.15					-0.0018												3	45.83	-84.52	0.00	0.083
0.18			-0.0030		-0.0018												4	46.81	-83.63	0.89	0.054
0.21					-0.0018	-0.0625											4	46.42	-82.83	1.68	0.036
0.39					-0.0018	-0.2499		-0.0035						0.0037			6	49.71	-82.76	1.76	0.035
0.14					-0.0017			0.0001									4	46.24	-82.48	2.04	0.030
0.17			-0.0038		-0.0018			0.0002									5	47.80	-82.44	2.08	0.029
0.69					-0.0018		-0.2900										4	46.22	-82.43	2.08	0.029
0.40			-0.0036		-0.0018	-0.2326		-0.0034						0.0037			7	51.42	-82.25	2.26	0.027
0.18				-0.0006	-0.0017												4	46.06	-82.11	2.40	0.025
0.16		-0.0005			-0.0017												4	45.90	-81.80	2.72	0.021

Table S2.7 Summary results of the top model, with R^2 = 53%.

	Estimate	s.e.	t	p
Intercept	0.147	0.017	8.810	<0.001
Par	-0.002	0.000	-5.073	<0.001

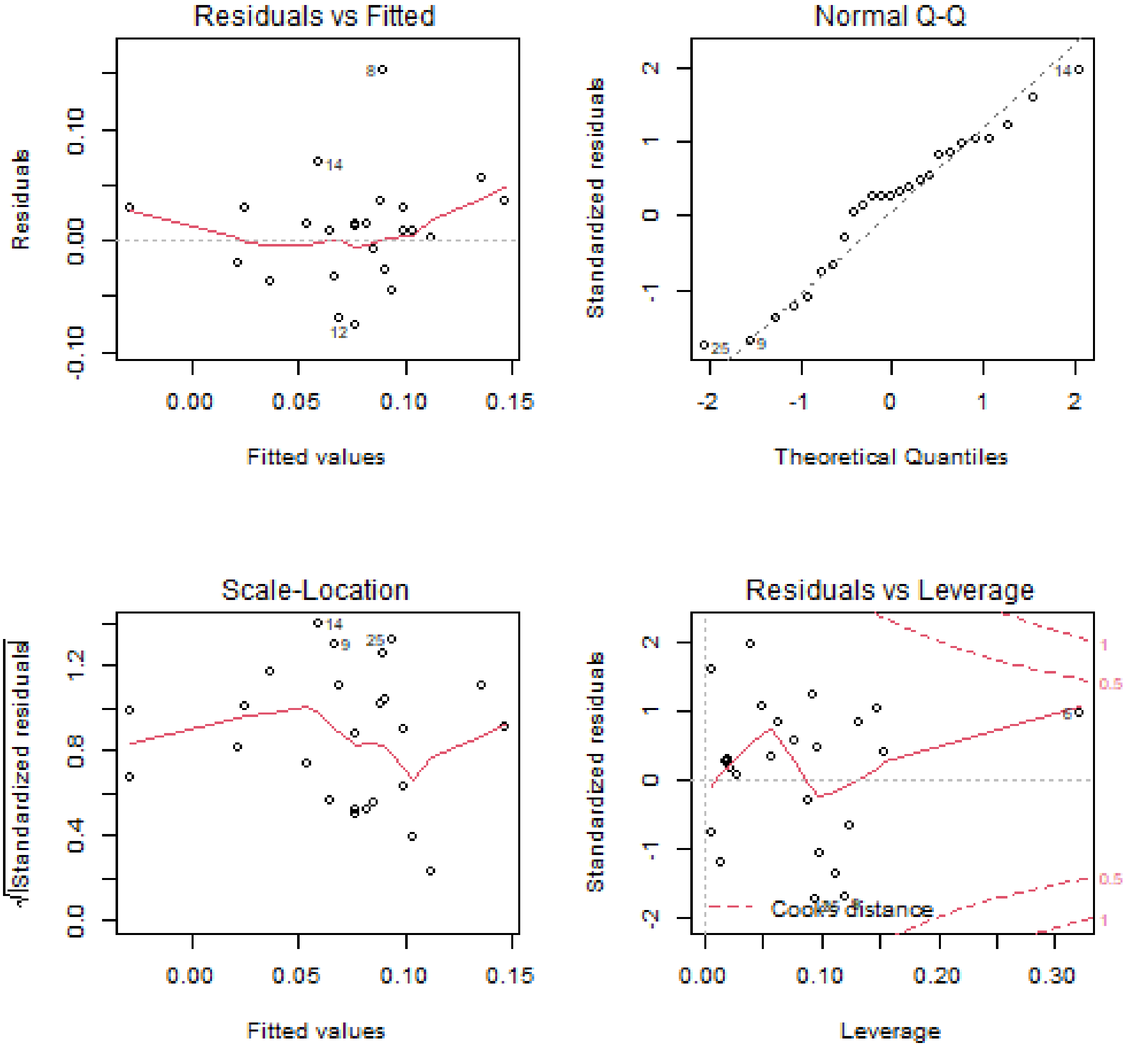

Figure S2.3 Plots of residuals of the top model for *C. flavipennella*.

Coleophora lutipennella:

Table S2.8. Top ten models for *C. lutipennella* abundance. See Table S2.2 for abbreviations.

Intercept	Cluster	Day	BB	Diam	Par	IH	Fcyt	PI	sdPI	Day:PI	BB:PI	Diam:PI	Par:PI	IH:PI	Fcyt:PI	PI:sdPI	df	logLik	AICc	delta	weight
0.07	+				-0.0007												4	47.49	-84.98	0.00	0.044
0.03	+		0.0034		-0.0007												5	48.91	-84.66	0.32	0.038
0.97	+				-0.0008		-0.4831										5	48.76	-84.36	0.62	0.032
0.05	+				-0.0007			0.0002									5	48.56	-83.97	1.01	0.027
0.88	+		0.0032		-0.0008		-0.4515										6	50.15	-83.63	1.35	0.022
0.11	+		0.0042		-0.0007	-0.0846											6	50.00	-83.33	1.65	0.019
0.00	+		0.0040														4	46.57	-83.14	1.84	0.018
0.03	+																3	44.97	-82.79	2.19	0.015
0.10	+			-0.0008	-0.0006												5	47.96	-82.76	2.22	0.015
0.21	+	-0.0024	0.0055			-0.1360											6	49.69	-82.71	2.27	0.014

Table S2.9 Summary results of the top model, with $R^2 = 41\%$.

	Estimate	s.e.	t	p
Intercept	0.067	0.019	3.472	0.002
Side = Zielonka	0.042	0.014	2.966	0.007
Par	-0.001	0.000	-2.218	0.037

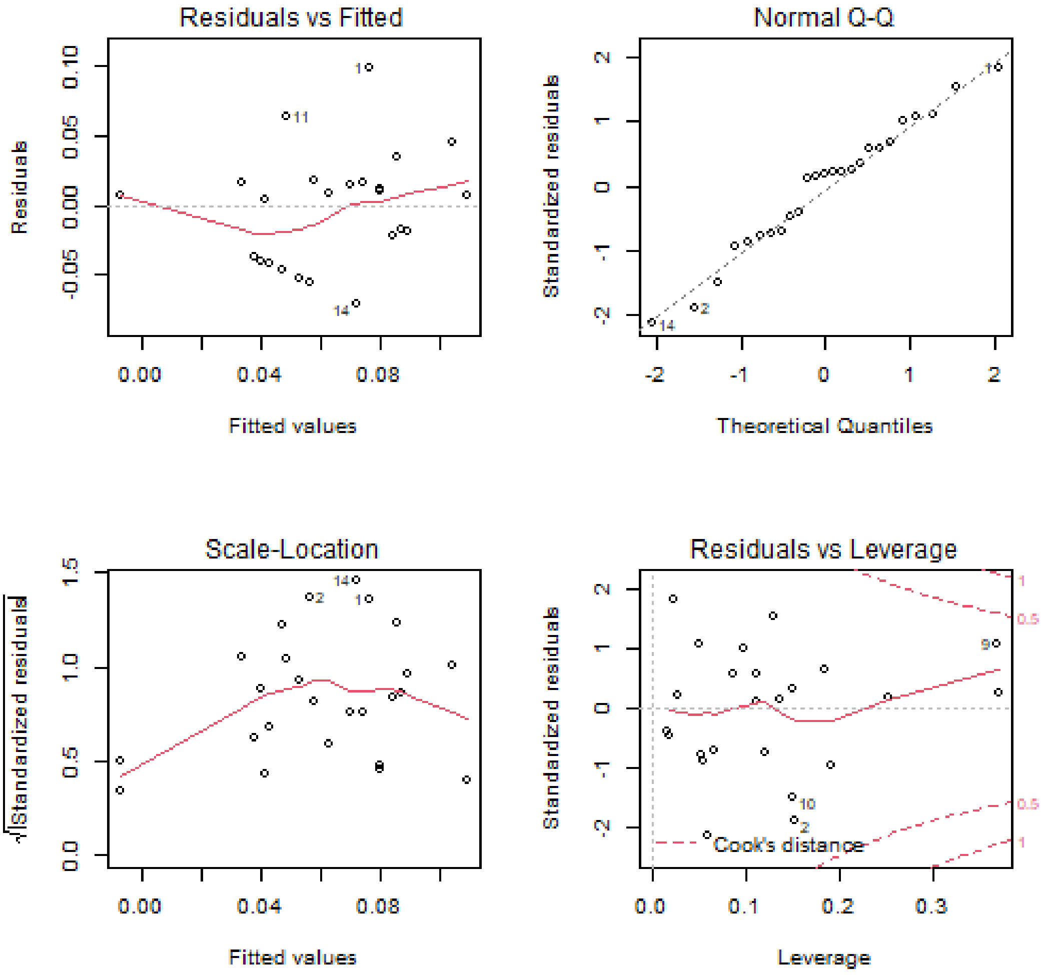

Figure S2.4 Plots of residuals of the top model for *C. lutipennella*.

Semi-concealed caterpillars:

Table S2.10. Top ten models for semi-concealed caterpillar abundance. See Table S2.2 for abbreviations.

Intercept	Cluster	Day	BB	Diam	Par	IH	Fcyt	PI	sdPI	Day:PI	BB:PI	Diam:PI	Par:PI	IH:PI	Fcyt:PI	PI:sdPI	df	logLik	AICc	delta	weight
0.12								0.0010									3	31.99	-56.84	0.00	0.085
0.25		-0.0031						0.0008									4	32.98	-55.97	0.87	0.055
0.01						0.1161		0.0010									4	32.66	-55.33	1.51	0.040
0.14	+							0.0010									4	32.46	-54.91	1.93	0.033
0.14					-0.0003			0.0009									4	32.17	-54.33	2.51	0.024
0.13								0.0010	-0.0001								4	32.05	-54.10	2.74	0.022
-0.07							0.1024	0.0010									4	32.01	-54.01	2.83	0.021
0.12			0.0002					0.0010									4	31.99	-53.99	2.85	0.020
0.12				0.0001				0.0010									4	31.99	-53.99	2.86	0.020
0.18		-0.0046		0.0023				0.0009									5	33.56	-53.96	2.88	0.020

Table S2.11 Summary results of the top model, with $R^2 = 34\%$. Exclusion of outliers 11 and 15 does not affect this result qualitatively.

	Estimate	s.e.	t	p
Intercept	0.124	0.022	5.778	<0.001
PI	0.001	0.000	3.411	0.002

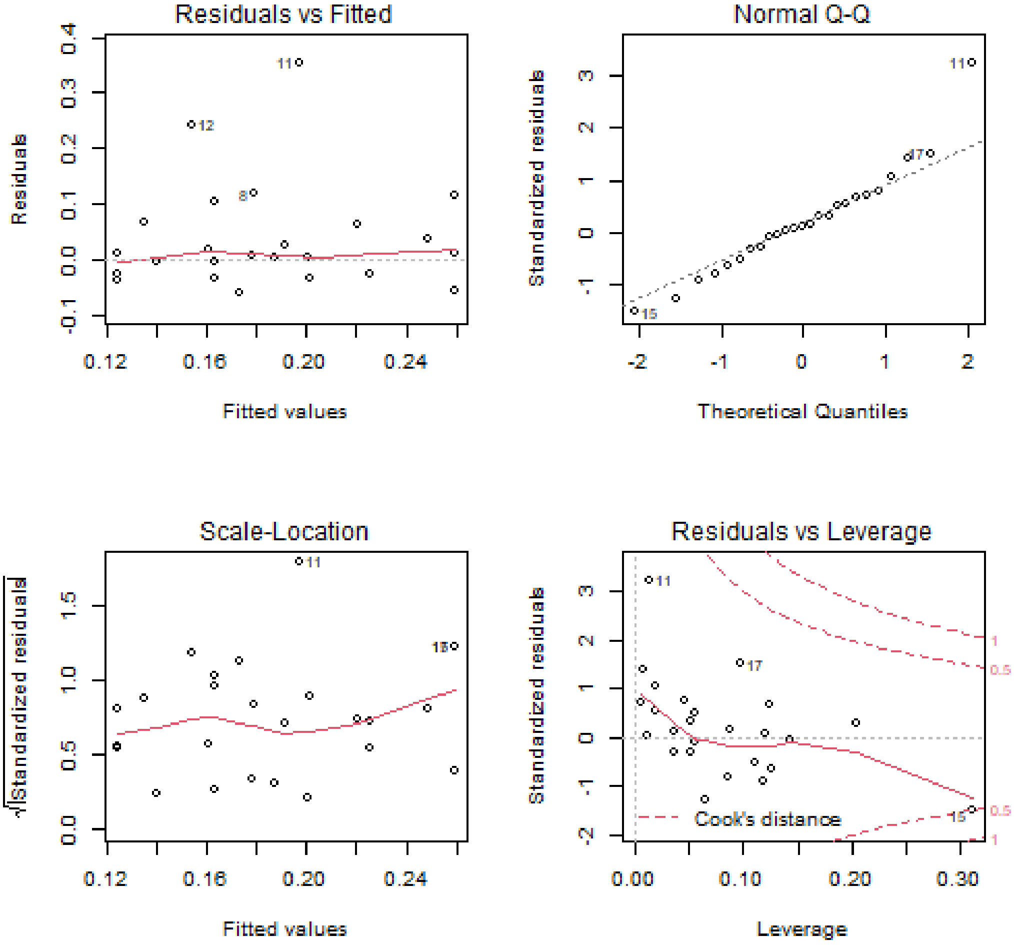

Figure S2.5 Plots of residuals of the top model for semi-concealed caterpillars.

Carcina quercana:

Table S2.12. Top ten models for *C. quercana* caterpillar abundance. See Table S2.2 for abbreviations.

Intercept	Cluster	Day	BB	Diam	Par	IH	Fcyt	PI	sdPI	Day:PI	BB:PI	Diam:PI	Par:PI	IH:PI	Fcyt:PI	PI:sdPI	df	logLik	AICc	delta	weight
0.09					-0.0006												3	44.62	-82.10	0.00	0.053
0.06																	2	43.10	-81.66	0.44	0.043
0.03			0.0034														3	44.12	-81.09	1.01	0.032
0.05								0.0002									3	44.08	-81.01	1.09	0.031
0.06			0.0029		-0.0006												4	45.42	-80.85	1.25	0.028
0.07					-0.0006			0.0002									4	45.37	-80.73	1.36	0.027
0.08					-0.0007				0.0002								4	44.91	-79.81	2.29	0.017
0.10				-0.0008													3	43.46	-79.78	2.32	0.017
0.07		0.0006			-0.0007												4	44.74	-79.47	2.63	0.014
0.02			0.0027					0.0002									4	44.68	-79.37	2.73	0.014

Table S2.13 Summary results of the top model, with $R^2 = 11\%$.

	Estimate	s.e.	t	p
Intercept	0.088	0.018	4.990	<0.001
Par	-0.001	0.000	-1.723	0.098

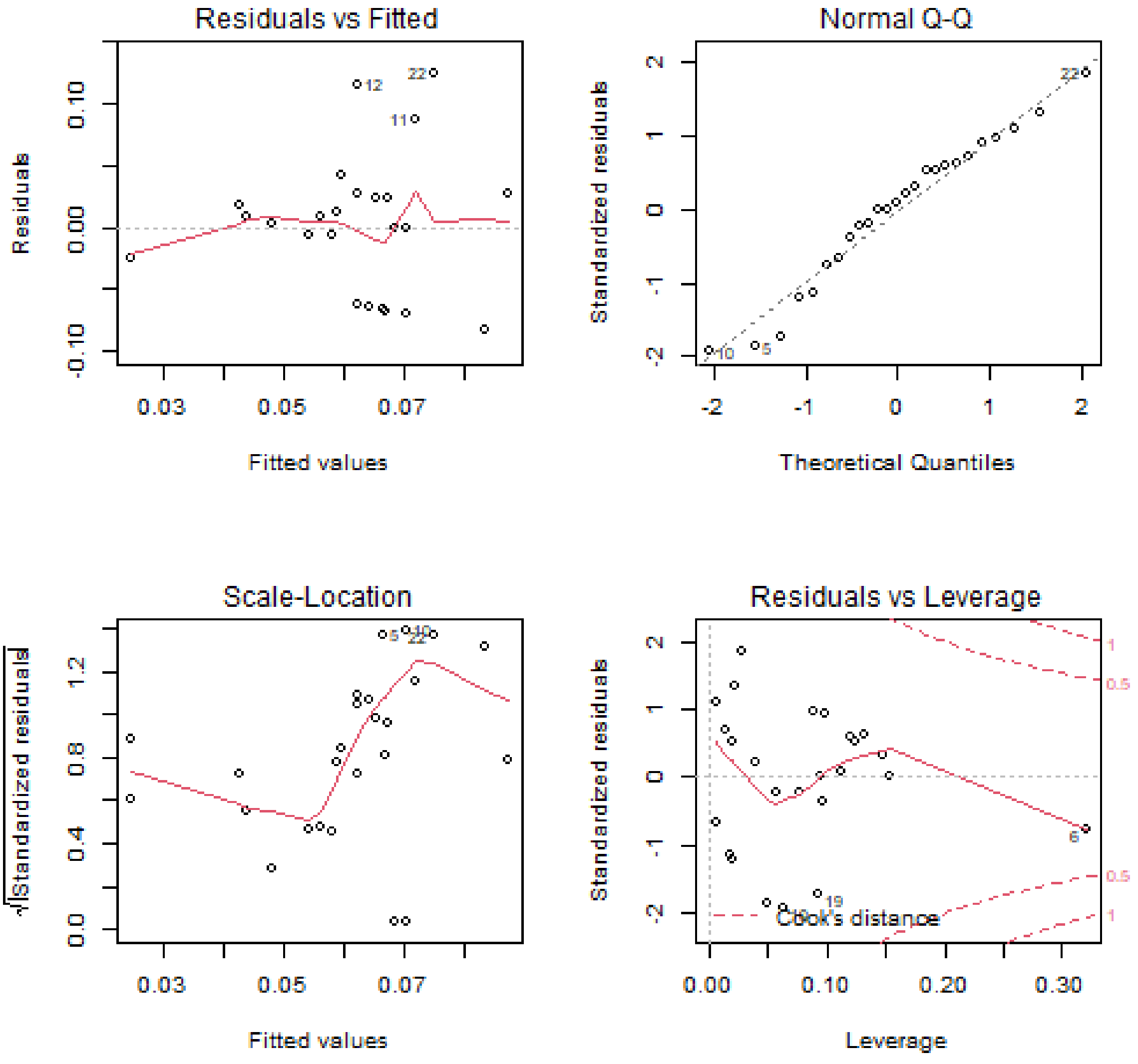

Figure S2.6 Plots of residuals of the top model for *C. quercana* caterpillars.

Free-living caterpillars:

Table S2.14. Top ten models for free-living caterpillar abundance. See Table S2.2 for abbreviations.

Intercept	Cluster	Day	BB	Diam	Par	IH	Fcyt	PI	sdPI	Day:PI	BB:PI	Diam:PI	Par:PI	IH:PI	Fcyt:PI	PI:sdPI	df	logLik	AICc	delta	weight
0.36		-0.0046						0.0006									4	33.28	-56.56	0.00	0.084
0.18								0.0009									3	31.09	-55.03	1.53	0.039
0.47		-0.0065															3	30.96	-54.78	1.78	0.035
0.37	+	-0.0045						0.0006									5	33.57	-53.97	2.58	0.023
0.33		-0.0054		0.0012				0.0007									5	33.43	-53.71	2.85	0.020
0.37		-0.0043			-0.0003			0.0006									5	33.41	-53.67	2.89	0.020
0.05						0.1258		0.0009									4	31.82	-53.65	2.91	0.020
0.30		-0.0042				0.0469		0.0007									5	33.38	-53.60	2.96	0.019
-0.08		-0.0047					0.2377	0.0007									5	33.37	-53.57	2.99	0.019
0.37		-0.0045	-0.0011					0.0007									5	33.32	-53.49	3.07	0.018

Table S2.15 Summary results of the top model, with R^2 = 40%. Removing outlier no 11 or 15 does not qualitatively change these results.

	Estimate	s.e.	t	p
Intercept	0.363	0.092	3.939	0.001
Day	-0.005	0.002	-2.054	0.052
PI	0.001	0.000	2.116	0.046

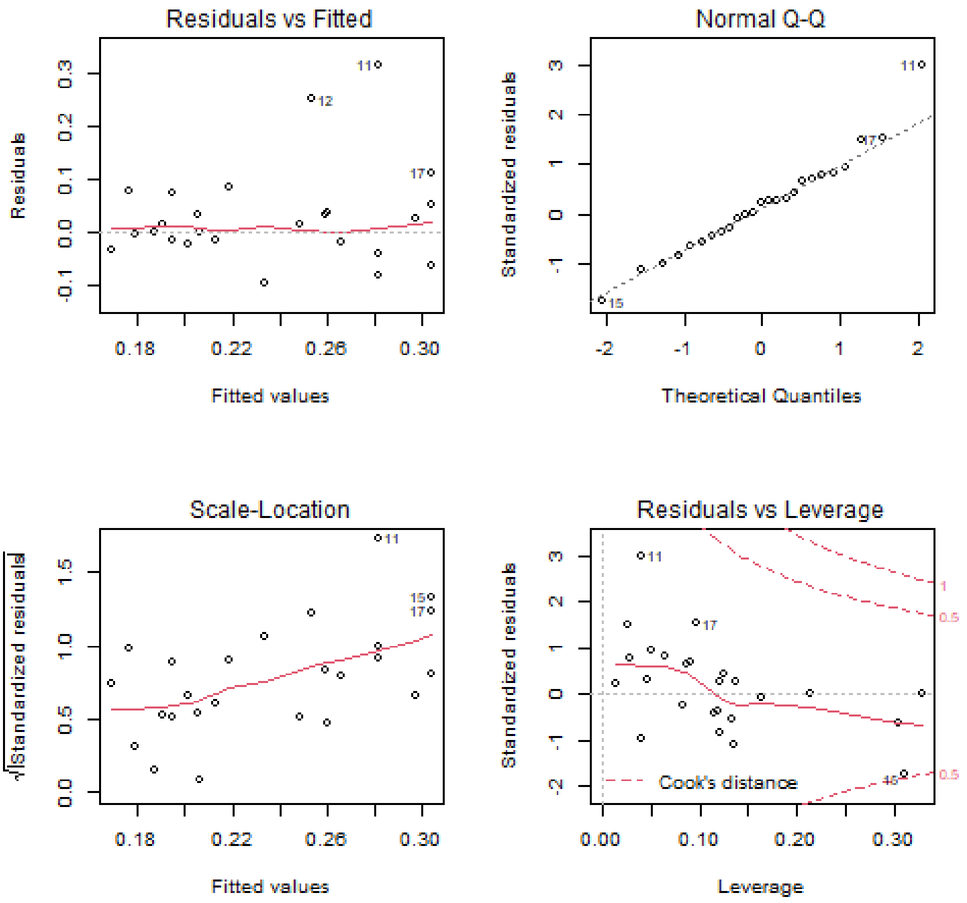

Figure S2.7. Plots of residuals of the top model for free-living caterpillars.

Geometrids:

Table S2.16. Top ten models for geometrid caterpillar abundance. See Table S2.2 for abbreviations.

Intercept	Cluster	Day	BB	Diam	Par	IH	Fcyt	PI	sdPI	Day:PI	BB:PI	Diam:PI	Par:PI	IH:PI	Fcyt:PI	PI:sdPI	df	logLik	AICc	delta	weight
0.09					-0.0009				0.0008								4	41.44	-72.88	0.00	0.039
0.06	+				-0.0010				0.0010								5	43.02	-72.87	0.01	0.038
0.17				-0.0022					0.0005								4	41.12	-72.25	0.63	0.028
0.27			-0.0049	-0.0028													4	40.96	-71.93	0.95	0.024
0.21				-0.0026													3	39.39	-71.64	1.24	0.021
0.12	+	-0.0020			-0.0008				0.0010								6	44.06	-71.45	1.43	0.019
0.12	+	-0.0028							0.0008								5	42.30	-71.44	1.45	0.019
0.16				-0.0015	-0.0007				0.0007								5	42.27	-71.38	1.50	0.018
0.15		-0.0026							0.0006								4	40.69	-71.38	1.50	0.018
0.05									0.0006								3	39.18	-71.22	1.66	0.017

Table S2.17 Summary results of the top model with R^2 = 30%. Removing outlier no 18 does not change this result qualitatively (without no 18: sdPI p = 0.017, parasitism p = 0.005).

	Estimate	s.e.	t	p
Intercept	0.088	0.021	4.085	<0.001
sdPI	0.001	0.000	2.728	0.012
Par	-0.001	0.000	-2.087	0.049

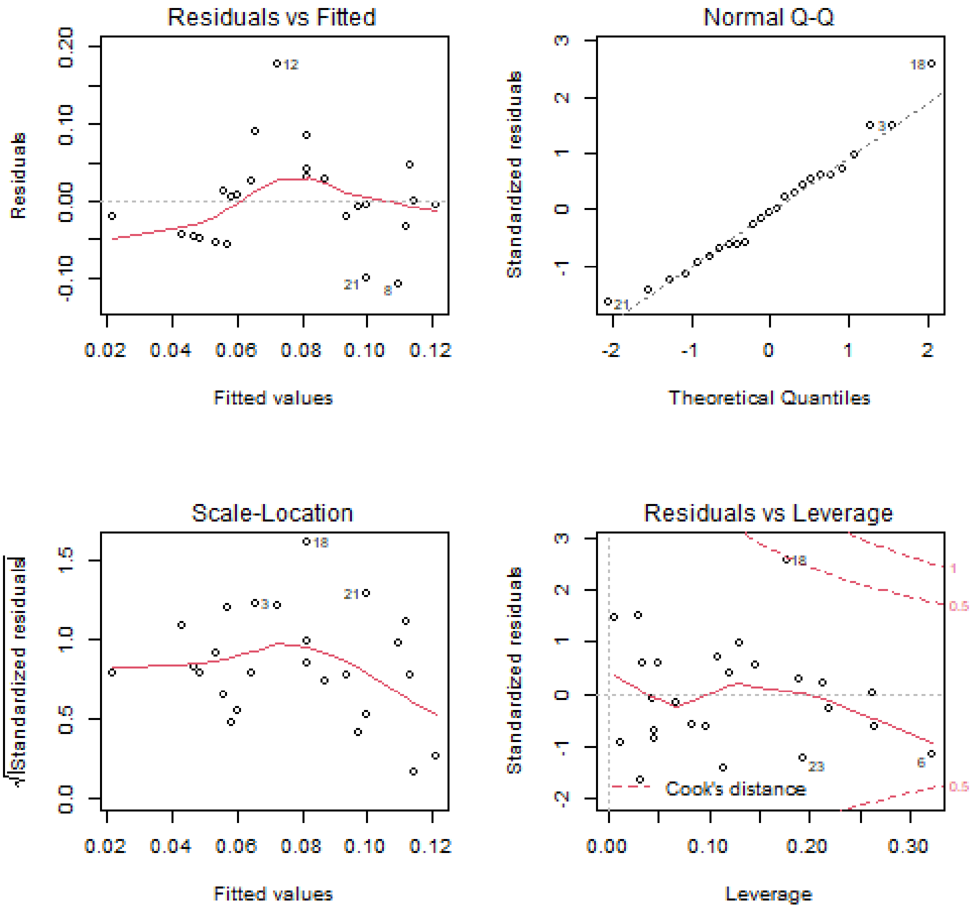

Figure S2.8. Plots of residuals of the top model for geometrid caterpillars.

Parasitism rate:

Table S2.18. Top ten models for parasitism rate. See Table S2.2 for abbreviations.

Intercept	Cluster	Day	BB	Diam	Par	IH	Fcyt	PI	sdPI	Day:PI	BB:PI	Diam:PI	Par:PI	IH:PI	Fcyt:PI	PI:sdPI	df	logLik	AICc	delta	weight
-21.38				0.9979					0.2905								4	-110.38	230.77	0.00	0.062
-46.25	+			1.2103					0.3893								5	-109.07	231.29	0.52	0.047
27.21									0.2918								3	-112.51	232.17	1.40	0.031
39.31								-0.1335	0.2478								4	-111.26	232.51	1.74	0.026
-10.08		1.0656							0.2850								4	-111.34	232.68	1.92	0.024
-7.23			-0.8715	0.9356					0.2387								5	-109.99	233.14	2.38	0.019
279.24				1.1196			-164		0.2427								5	-110.04	233.23	2.46	0.018
-111.55	+			1.4287		47.7884			0.4541								6	-108.31	233.29	2.53	0.017
46.91								-0.2153	-0.1859							0.0058	5	-110.08	233.31	2.54	0.017
13.84			-1.7390	0.8777													4	-111.69	233.38	2.62	0.017

Table S2.19 Summary results of the top model, with $R^2 = 31\%$.

	Estimate	s.e.	t	p
Intercept	-21.4	24.8	-0.863	0.398
sdPI	0.291	0.120	2.421	0.024
Diameter	0.998	0.494	2.021	0.056

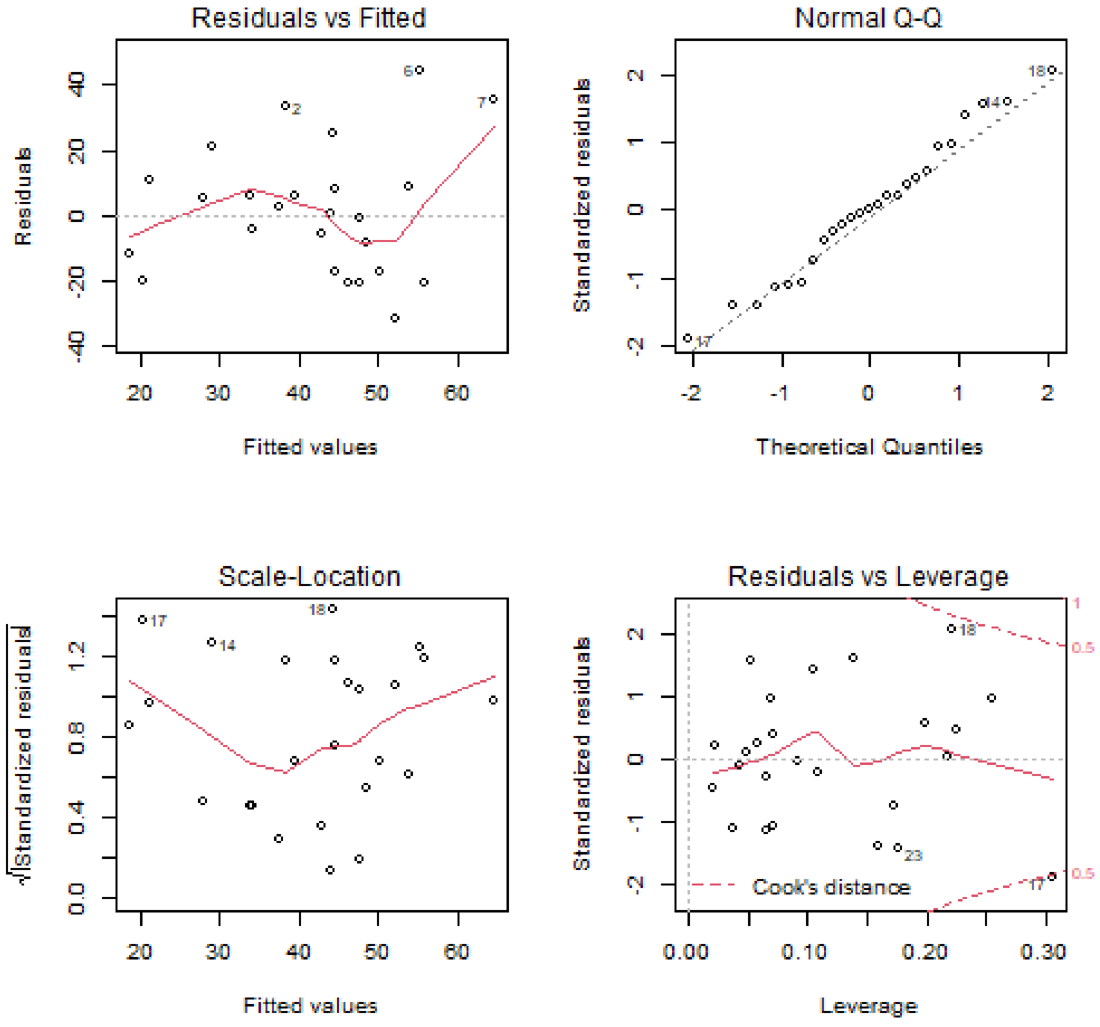

Figure S2.9. Plots of residuals of the top model for the parasitism rate.

Simpson Diversity:

Table S2.20. Top ten models for Simpson diversity. See Table S2.2 for abbreviations.

Intercept	Cluster	Day	BB	Diam	Par	IH	Fcyt	PI	sdPI	Day:PI	BB:PI	Diam:PI	Par:PI	IH:PI	Fcyt:PI	PI:sdPI	df	logLik	AICc	delta	weight
0.92	+			0.021				0.023				-0.0005					7	18.35	-15.24	0.00	0.10
0.79	+			0.027	0.291	-1.550		0.029				-0.0006					8	20.12	-13.95	1.29	0.05
1.65	+			0.014	0.371	-1.631		0.021	-0.003			-0.0005					9	22.66	-13.46	1.77	0.04
7.08	+	0.038		0.022		-1.339	-4.073	-0.086		-0.0006		-0.0005			0.069		11	29.60	-13.20	2.04	0.04
-0.35	+	0.030		0.029		-1.440		0.043		-0.0004		-0.0006					9	22.37	-12.89	2.35	0.03
1.58	+					-0.800			-0.004								5	13.18	-12.83	2.41	0.03
1.57	+			0.011		-1.350		0.016	-0.002			-0.0004					8	19.49	-12.70	2.54	0.03
1.17	+			0.025		-1.763		0.017				-0.0005		0.0075			8	19.45	-12.61	2.63	0.03
9.41						-0.621	-4.408	-0.143	-0.002						0.0767		7	17.00	-12.53	2.71	0.03
0.97	+		0.007	0.020		-1.390		0.023				-0.0005					8	19.12	-11.95	3.29	0.02

Table S2.21 Summary results of the second best model with R^2 = 43%.

	Estimate	s.e.	t	p
Intercept	0.247	0.378	0.654	0.521
Diameter	0.020	0.006	3.166	0.005
IH	-0.719	0.285	-2.522	0.021
PI	0.017	0.004	3.803	0.001
Diameter*PI	0.000	0.000	-3.975	0.001

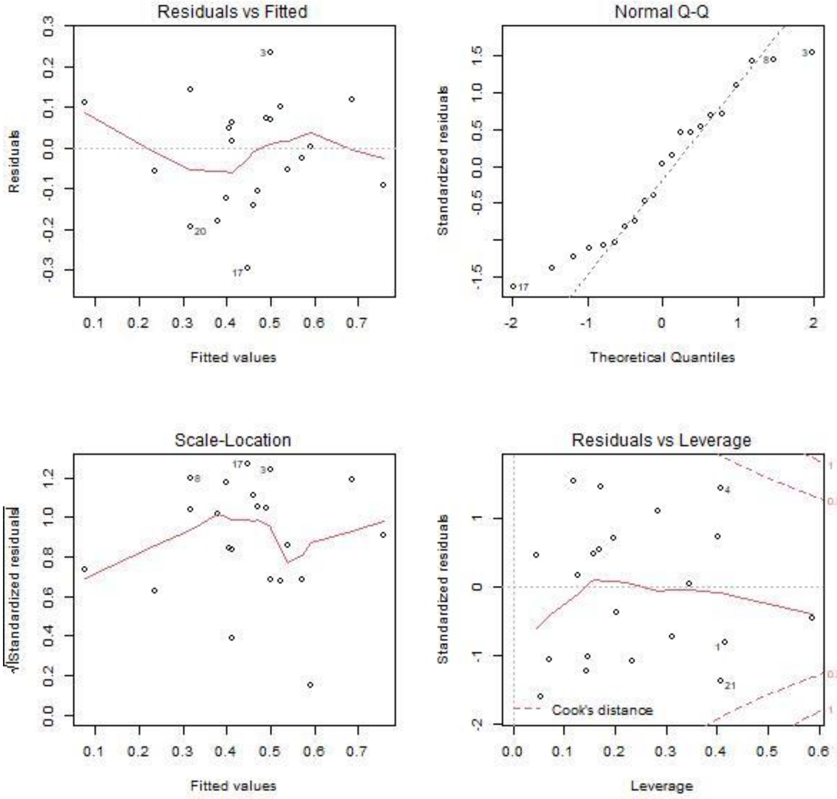

Figure S2.10. Plots of residuals of the second-best model for Simpson diversity. Exclusion of one or two outliers does not change the results qualitatively.

Community weighted wingspan:

Table S2.22. Top ten models for community weighted wingspan. See Table S2.2 for abbreviations.

(Intercept)	Cluster	DayNoSam	BB	Diameter	pPar	IH	Fcyt	PI	sdPI	yNoSamp	BB:PI	Diameter:F	PI:pPar	IH:PI	Fcyt:PI	PI:sdPI	df	logLik	AICc	delta	weight
16.90	+																3	-47.23	101.72	0.00	0.04
20.23		-0.119															3	-47.33	101.93	0.22	0.04
19.99	+	-0.093															4	-46.14	102.51	0.79	0.03
16.06																	2	-49.02	102.63	0.91	0.03
20.26	+			-0.060													4	-46.33	102.89	1.17	0.02
15.94	+		0.110														4	-46.47	103.17	1.45	0.02
16.87					-2.634												3	-48.13	103.51	1.80	0.02
20.51		-0.137							0.012								4	-46.77	103.76	2.04	0.02
16.66	+							0.006									4	-46.90	104.02	2.31	0.01
12.53						3.533											3	-48.42	104.10	2.38	0.01

Table S2.23 Summary results of the second best model with R^2 = 10%. The selected model was the NULL model.

	Estimate	s.e.	t	p
Intercept	16.9048	0.5762	29.34	<0.001
Cluster=Zielonka	-1.3894	0.7392	-1.88	0.0741

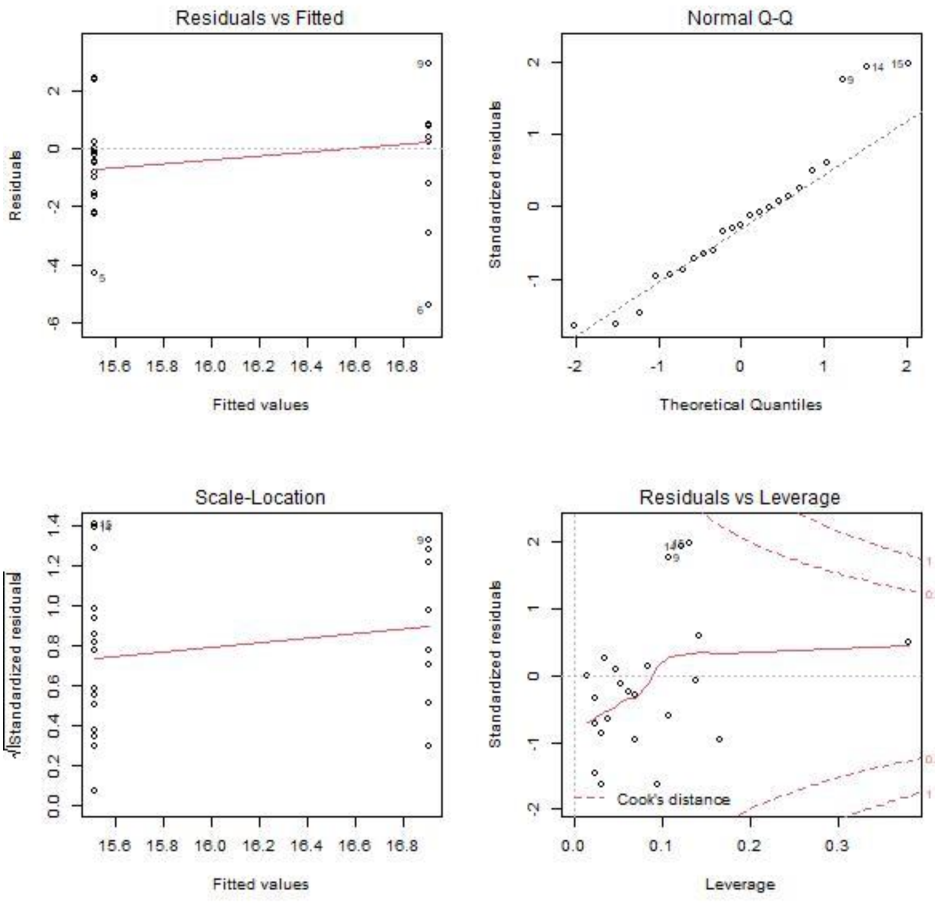

Figure S2.11. Plots of residuals of the second-best model for community weighted average wingspan.

Proportion of host-plant specialists:

Table S2.24. Top ten models for the proportion of host-plant specialists. See Table S2.2 for abbreviations.

(Intercept)	Cluster	ayNoSam	BB	Diameter	pPar	IH	Fcyt	PI	sdPI	yNoSamp	BB:PI	iameter:F	PI:pPar	IH:PI	Fcyt:PI	PI:sdPI	df	logLik	AICc	delta	weight
0.51					-0.309												3	7.67	-8.08	0.00	0.04
0.42																	2	6.15	-7.69	0.39	0.03
-0.05						0.466											3	7.45	-7.63	0.45	0.03
4.20							-2.050										3	7.34	-7.42	0.66	0.03
0.48	+																3	7.22	-7.17	0.91	0.02
0.69		-0.008															3	6.98	-6.71	1.37	0.02
0.50					-0.411				0.001								4	8.45	-6.69	1.39	0.02
0.16					-0.237	0.329											4	8.30	-6.38	1.70	0.02
0.50			-0.008														3	6.63	-6.00	2.08	0.01
0.71				-0.004	-0.367												4	8.10	-5.97	2.11	0.01

Table S2.25 Summary results of the second best model with $R^2 = 8\%$.

	Estimate	s.e.	t	p
Intercept	0.511	0.064	7.950	<0.001
Parasitism	-0.309	0.179	-1.726	0.099

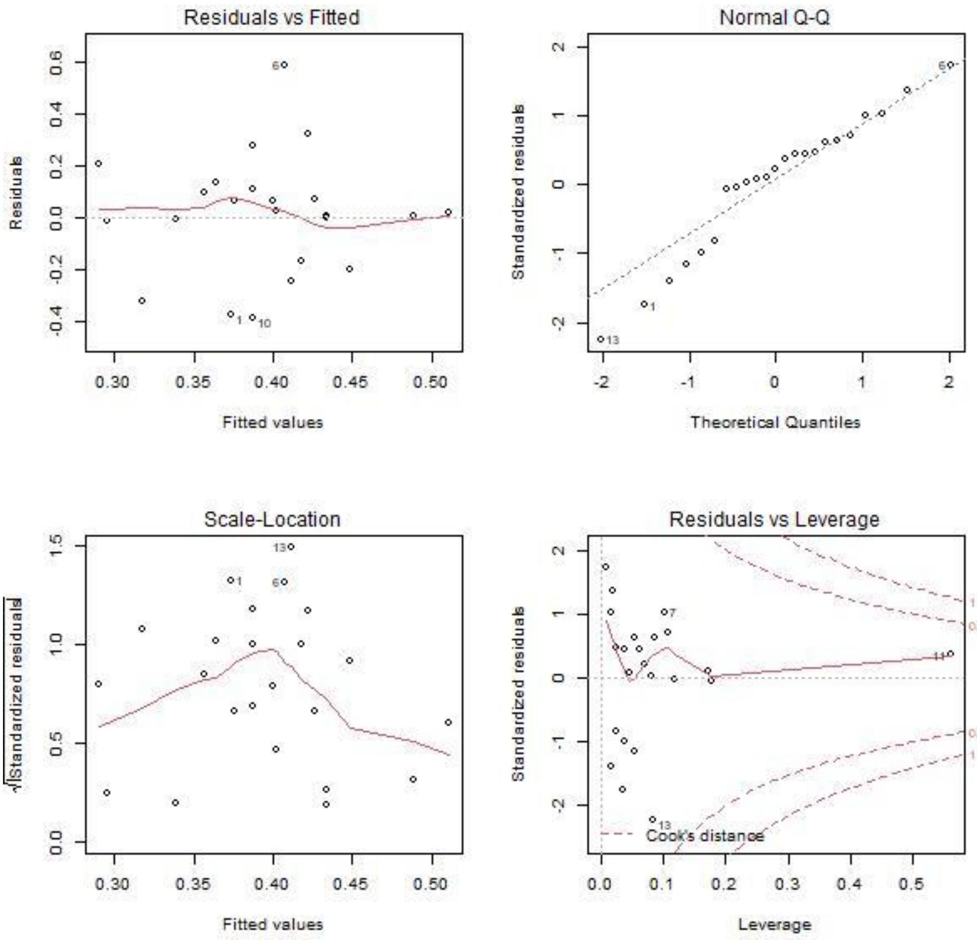

Figure S2.12. Plots of residuals of the second-best model for the proportion of host specialists.

Proportion of particular host-plant specialists:

Table S2.26. Top ten models for community weighted proportion of particular host-plant specialists. See Table S2.2 for abbreviations.

(Intercept	Cluster	ayNoSam	BB	Diameter	pPar	IH	Fcyt	PI	sdPI	yNoSamp	BB:PI	iameter:F	PI:pPar	IH:PI	Fcyt:PI	PI:sdPI	df	logLik	AICc	delta	weight
0.68																	2	10.87	-17.14	0.00	0.06
0.63	+																3	11.83	-16.40	0.74	0.04
0.81				-0.003													3	11.23	-15.20	1.94	0.02
0.81		-0.004															3	11.17	-15.07	2.07	0.02
0.83	+	-0.006															4	12.60	-14.98	2.16	0.02
1.22				-0.009				-0.001									4	12.57	-14.93	2.21	0.02
3.13	+						-1.361										4	12.57	-14.91	2.22	0.02
0.67	+				-0.183												4	12.53	-14.83	2.30	0.02
1.88							-0.650										3	11.04	-14.82	2.32	0.02
0.79						-0.116											3	10.98	-14.70	2.43	0.02

Table S2.27 Summary results of the second best model, with $R^2 = 4\%$.

	Estimate	s.e.	t	p
Intercept	0.631	0.044	14.276	<0.001
Cluster=Zielonka	0.077	0.057	1.353	0.190

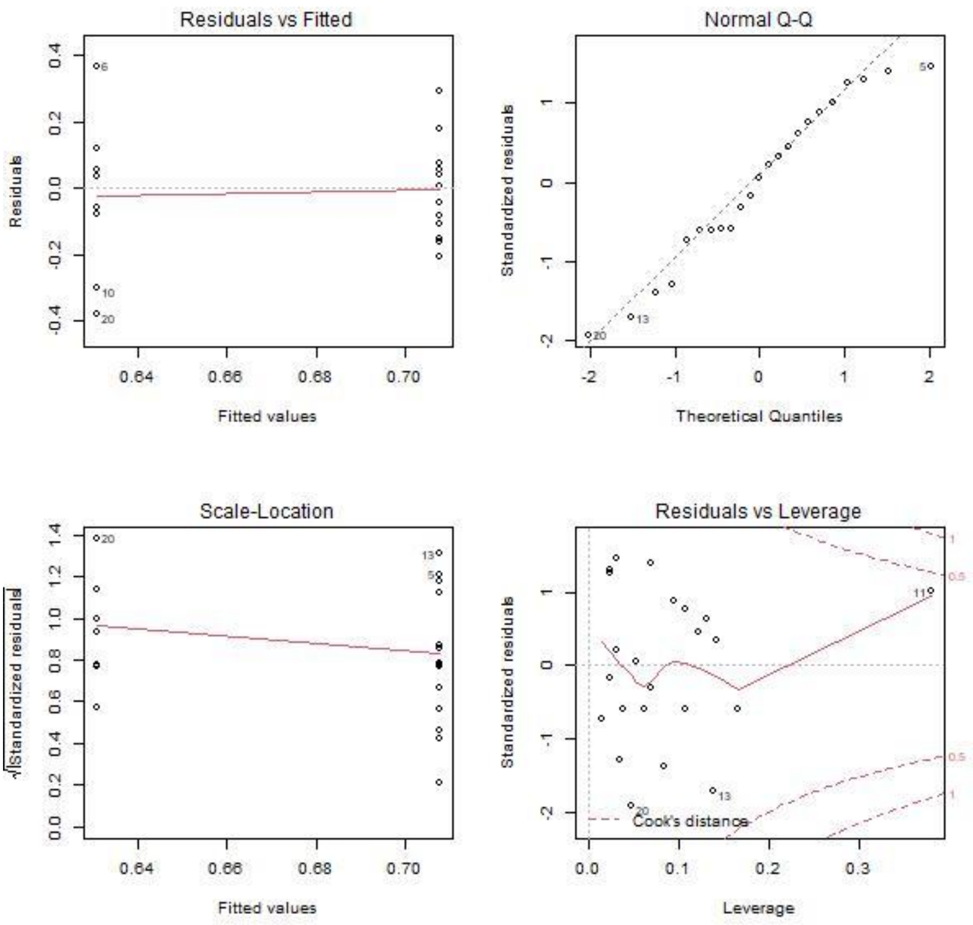

Figure S2.13. Plots of residuals of the second-best model for the proportion of particular host-plant specialists.

Reconstructed abundance and parasitism:

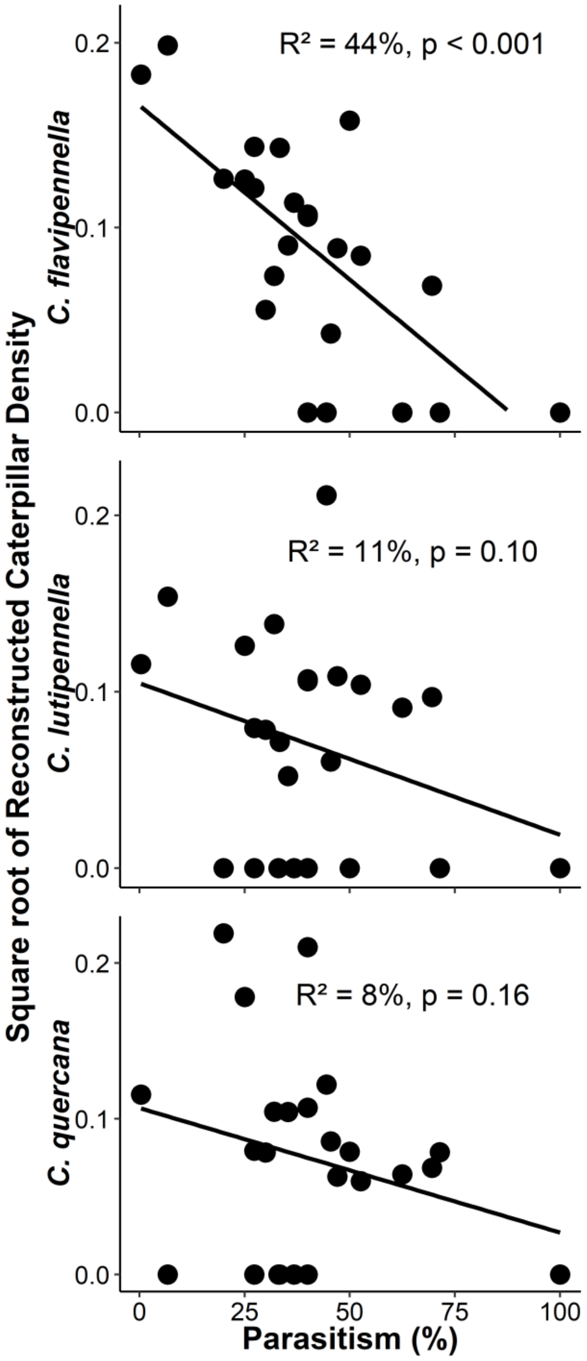

Figure S2.14. Relationships between the reconstructed abundance of the three most common caterpillar species and overall parasitism on a tree. For each species and for each tree, reconstructed abundance was calculated as: ('the number of adults' * (1+'overall parasitism rate on the tree')) and this reconstructed density was then square root transformed for data analyses and plotting. Results of OLS regression analyses are indicated in each plot. Since these results are similar to those using raw abundances (Figure 4), this indicates that the effect of parasitism on the abundance of moths reared from caterpillars is not due to an artefact of parasitism directly reducing the number of successfully reared adults.

Supplementary File S3

Tree traits and caterpillar communities

Table S3.1: Sampling details and characteristics of focal trees. Trees were chosen in two main clusters near Zielonka (Ziel) and Kamińsko (Kam) villages, and then in groups of 2-3 trees (except tree 17 which was single, because the other tree in the pair was initially misidentified due to severe leaf deformation). Leaf mass (dry weight in grams) was used to calculate caterpillar density. Budburst = Day of 50% of budburst, Diameter = Trunk diameter at breast height (DBH). IH = Standardized Individual Heterozygosity, and Fcyt = Genome size (see Appendix 1). PI = Phylogenetic Isolation, calculated as the average phylogenetic crown age of the neighbouring trees, with the standard deviation (sdPI) representing the Phylogenetic Heterogeneity of the neighbourhood.

	Location:		Sampling:		Development:		Genetic traits:		Neighbourhood:	
Tree	Cluster	Group	Date	Leaf mass	Budburst	Diameter	IH	Fcyt	PI	sdPI
1	Ziel	ZielBig1	5/31/2019	97.036	5/3/2019	65.6	0.80	1.894	40	0
2	Ziel	ZielBig1	5/31/2019	278.247	4/28/2019	59.8	0.95	1.875	40	0
3	Ziel	ZielBig1	5/31/2019	121.991	4/25/2019	49.7	0.91	1.833	11	20
4	Ziel	ZielBig2	5/31/2019	424.248	4/28/2019	59.8	0.91	1.869	16	22
5	Ziel	ZielBig2	5/31/2019	260.299	4/30/2019	49.3	0.91	1.857	0	0
13	Kam	PławnoBig	6/3/2019	234.454	4/25/2019	58.6	0.91	1.842	55	63
15	Kam	PławnoBig	6/3/2019	70.852	5/2/2019	65.9	1.14	1.835	79	70
17	Kam	Ka16	5/19/2019	33.974	5/2/2019	51.9	1.25	1.852	56	69
22	Kam	KaPin2	5/19/2019	795.934	4/28/2019	40.4	1.14	1.852	66	71
23	Kam	KaPin2	5/19/2019	246.443	4/22/2019	56	1.02	1.9	38	46
24	Kam	KaHB	5/17/2019	78.654	4/27/2019	49	1.02	1.825	75	64
25	Kam	KaHB	5/17/2019	31.655	4/28/2019	61.8	1.14	1.842	31	29
26	Kam	KaHB	5/17/2019	106	4/26/2019	64.3	1.14	1.808	0	0
27	Ziel	ZielWN1	5/21/2019	240.784	4/26/2019	41.7	1.07	1.861	129	30
28	Ziel	ZielWN2	5/21/2019	483.059	5/2/2019	42.7	0.91	1.854	140	0
30	Ziel	ZielWN2	5/27/2019	604.255	4/25/2019	46.2	0.80	1.862	40	68
31	Ziel	ZielRoadS	5/21/2019	150.132	5/8/2019	41.7	1.02	1.867	140	0
32	Ziel	ZielRoadS	5/21/2019	360.707	4/26/2019	43.6	0.91	1.83	70	77
33	Ziel	ZielRoadS	5/21/2019	135.153	4/24/2019	40.1	1.02	1.837	140	0
34	Ziel	ZielRoadN	5/21/2019	372.35	4/25/2019	50.3	0.86	1.817	105	65
35	Ziel	ZielRoadN	5/21/2019	124.799	5/5/2019	40.1	1.02	1.796	105	65
37	Kam	KaPin1	6/3/2019	74.968	4/29/2019	53.8	1.02	1.839	100	68
38	Kam	KaPin1	6/3/2019	496.41	4/28/2019	55.7	0.91	1.849	80	75
44	Ziel	ZielBig2	5/31/2019	392.806	4/30/2019	55.7	1.07	1.848	51	68
45	Ziel	ZielWN1	5/27/2019	422.343	4/28/2019	55.7	1.14	1.873	0	0

Table S3.2. Number of caterpillars sampled from each tree per functional group, with the total number of herbivores and parasitoids reared to adult, and the Simpson diversity of caterpillars reared to adulthood (plus some case bearers identified from cases). On tree 26, the number of identified moths is greater than caterpillar abundance because caterpillar abundance from one branch was excluded from analysis (see main text).

	Caterpillar abundance:				Reared adults:		Identified	Diversity	Functional traits:		
Tree	Casebearers	Semi-concealed	Free-living	Geometridae	Moths	Parasitoids	Moths	Simpson	Wingspan	p. Spec	p. Part Spec.
1	5	7	0	0	5	4	5	0.67	14.0	0.00	0.60
2	8	7	2	0	2	5	2	0.63	15.5	0.50	0.50
3	6	5	3	3	3	2	4	0.73	13.3	0.50	0.75
4	15	8	5	2	8	10	9	0.80	15.0	0.33	0.67
5	6	2	3	0	5	2	4	0.57	11.3	0.75	1.00
13	4	8	2	0	0	5	0	.	.	.	.
15	3	3	1	0	0	3	0	.	.	.	.
17	3	3	0	0	2	1	2	0.19	11.5	1.00	1.00
22	12	29	20	7	11	10	12	0.55	17.8	0.50	0.67
23	5	8	9	2	8	3	8	0.43	14.0	0.50	0.75
24	6	24	4	2	9	3	9	0.46	19.8	0.44	0.56
25	2	5	3	2	3	2	3	0.20	17.7	0.00	0.33
26	1	1	1	0	2	0	32	0.75	17.3	0.53	0.69
27	9	20	5	2	11	11	11	0.47	15.7	0.45	0.55
28	18	20	8	2	17	8	18	0.59	15.4	0.17	0.56
30	22	10	14	8	18	6	16	0.55	17.9	0.44	0.75
31	22	21	5	2	18	10	17	0.57	17.9	0.47	0.76
32	11	17	14	10	7	16	7	0.32	15.3	0.29	0.71
33	12	10	7	2	13	1	14	0.48	14.7	0.50	0.79
34	16	15	7	2	9	7	9	0.37	13.9	0.44	0.89
35	5	5	0	0	3	2	3	0.15	14.5	0.67	1.00
37	2	6	1	1	4	1	4	0.18	17.1	0.25	0.25
38	11	14	7	3	11	6	14	0.45	15.7	0.43	0.57
44	6	5	8	5	3	5	3	0.12	13.3	0.00	0.67
45	4	8	7	2	7	3	8	0.27	15.1	0.25	0.63

Table S3.3: Abundance of reared adults and those identified from cases, per species, and tree.

**Group**	**Family**	**Species**	**Total**	**Tree**																								
**Casbearers**				1	2	3	4	5	13	15	17	22	23	24	25	26	27	28	30	31	32	33	34	35	37	38	44	45
	Coleophoridae	*Coleophora flavipennella* (Dup.)	53	0	0	1	2	3	0	0	2	1	4	1	0	8	5	2	6	4	1	5	2	1	1	3	0	1
		C. lutipennella (Zell.)	42	3	0	1	3	1	0	0	0	2	1	1	0	3	0	7	3	2	2	4	3	1	0	1	2	2
		C. ibipennella Zell.	5	0	1	1	1	0	0	0	0	0	0	0	0	0	0	0	0	0	0	0	1	0	0	1	0	0
		C. kuehnella (Goeze)	5	0	0	0	0	0	0	0	0	1	0	0	0	1	0	0	0	0	0	0	0	0	0	2	0	1
		C.anatipenella (Hüb.)	1	0	0	0	0	0	0	0	0	0	0	0	0	0	0	1	0	0	0	0	0	0	0	0	0	0
**Semi-concealed**																												
	Peleopodidae	*Carcina quercana* (Fab.)	38	1	1	1	1	0	0	0	0	4	0	2	1	5	1	4	3	2	1	0	1	0	3	4	1	2
	Nolidae	*Nycteola revayana* (Scop.)	2	0	0	0	1	0	0	0	0	0	0	0	0	0	1	0	0	0	0	0	0	0	0	0	0	0
	Pyralidae	*Acrobasis repandana* (Fab.)	20	0	0	0	0	0	0	0	0	3	0	2	0	8	0	1	0	3	0	2	1	0	0	0	0	0
		A. tumidana (Den. & Schiff.)	5	0	0	0	0	0	0	0	0	1	0	1	0	0	0	0	0	1	1	0	0	1	0	0	0	0
		Phycita roborella (Den. & Schiff.)	5	0	0	0	1	0	0	0	0	0	1	0	0	0	0	1	0	1	0	1	0	0	0	0	0	0
	Tortricidae	*Archips xylosteana* (L.)	6	0	0	0	0	0	0	0	0	0	1	0	1	0	1	0	1	0	1	0	0	0	0	1	0	0
		A. podana (Scop.)	6	0	0	0	0	0	0	0	0	0	0	1	0	3	1	1	0	0	0	0	0	0	0	0	0	0
		A. crataegana (Hüb.)	1	0	0	0	0	0	0	0	0	0	0	0	0	0	0	0	0	0	0	0	0	0	0	1	0	0
		Eudemis profundana (Den. & Schiff.	9	0	0	0	0	0	0	0	0	0	0	0	1	2	1	0	0	2	1	0	1	0	0	0	0	1
		Pandemis corylana (Fab.)	5	1	0	0	0	0	0	0	0	0	0	0	0	1	0	1	0	1	0	0	0	0	0	1	0	0
		P.cerasana (Hüb.)	1	0	0	0	0	0	0	0	0	0	0	0	0	1	0	0	0	0	0	0	0	0	0	0	0	0
		Gypsonoma dealbana (Frölich)	1	0	0	0	0	0	0	0	0	0	0	0	0	0	0	0	0	1	0	0	0	0	0	0	0	0
	Ypsolophidae	*Ypsolopha ustella* (Cl.)	4	0	0	0	0	0	0	0	0	0	0	0	0	0	1	0	0	0	0	2	0	0	0	0	0	1
**Free-living**																												
	Erebidae	*Catocala sponsa* (L.)	1	0	0	0	0	0	0	0	0	0	0	0	0	0	0	0	1	0	0	0	0	0	0	0	0	0
		Orgyia antiqua (L.)	1	0	0	0	0	0	0	0	0	0	0	1	0	0	0	0	0	0	0	0	0	0	0	0	0	0
	Geometridae	*Chloroclysta siterata* (Huf.)	1	0	0	0	0	0	0	0	0	0	0	0	0	0	0	0	1	0	0	0	0	0	0	0	0	0
	Lycaenidae	*Favonius quercus* (L.)	2	0	0	0	0	0	0	0	0	0	1	0	0	0	0	0	1	0	0	0	0	0	0	0	0	0
